# Zebrafish Carrying *pycr1* Gene Deficiency Display Aging and Multiple Behavioral Abnormalities

**DOI:** 10.3390/cells8050453

**Published:** 2019-05-14

**Authors:** Sung-Tzu Liang, Gilbert Audira, Stevhen Juniardi, Jung-Ren Chen, Yu-Heng Lai, Zheng-Cai Du, Dar-Shong Lin, Chung-Der Hsiao

**Affiliations:** 1 Department of Bioscience Technology, Chung Yuan Christian University, Chung-Li 32023, Taiwan; stliang3@gmail.com (S.-T.L.); gilbertaudira@yahoo.com (G.A.); stvn.jun@gmail.com (S.J.); 2 Department of Chemistry, Chung Yuan Christian University, Chung-Li 32023, Taiwan; 3 Department of Biological Science & Technology, College of Medicine, I-Shou University, Kaohsiung 84001, Taiwan; jrchen@isu.edu.tw; 4 Department of Chemistry, Chinese Culture University, Taipei 11114, Taiwan; lyh21@ulive.pccu.edu.tw; 5 Guangxi Scientific Experimental Center of Traditional Chinese Medicine, Guangxi University of Chinese Medicine, Nanning 530200, China; 6 Guangxi Key Laboratory of Efficacy Study on Chinese Materia Medica, Guangxi University of Chinese Medicine, Nanning 530200, China; 7 Department of Pediatrics, Mackay Memorial Hospital, Taipei 252, Taiwan; 8 Department of Medical Research, Mackay Memorial Hospital, Taipei 252, Taiwan; 9 Department of Medicine, Mackay Medical College, New Taipei 252, Taiwan; 10 Center for Biomedical Technology, Chung Yuan Christian University, Chung-Li 32023, Taiwan; 11 Center for Nanotechnology, Chung Yuan Christian University, Chung-Li 32023, Taiwan

**Keywords:** zebrafish, TALEN, PYCR1, aging, disease model, behavioral alteration

## Abstract

Aging is a natural process that internal gene control and external stimuli mediate. Clinical data pointed out that homozygotic or heterozygotic mutation in the pyrroline-5-carboxylate reductase 1 (*PYCR1*) gene in humans caused cutis laxa (ARCL) disease, with progeroid appearance, lax and wrinkled skin, joint laxity, osteopenia, and mental retardation phenotypes. In this study, we aimed to generate *pycr1* knockout (KO) zebrafish and carried out biochemical characterizations and behavior analyses. Marked apoptosis and senescence were detected in *pycr1* KO zebrafish, which started from embryos/larvae stage. Biochemical assays showed that adult *pycr1* KO fish have significantly reduced proline and extracellular matrix contents, lowered energy, and diminished superoxide dismutase (SOD) and telomerase activity when compared to the wild type fish, which suggested the *pycr1* KO fish may have dysfunction in mitochondria. The *pycr1* KO fish were viable; however, displayed progeria-like phenotype from the 4 months old and reach 50% mortality around six months old. In adult stage, we found that *pycr1* KO fish showed reduced locomotion activity, aggression, predator avoidance, social interaction interest, as well as dysregulated color preference and circadian rhythm. In summary, we have identified multiple behavioral alterations in a novel fish model for aging with *pycr1* gene loss-of-function by behavioral tests. This animal model may not only provide a unique vertebrate model to screen potential anti-aging drugs in the future, but also be an excellent in vivo model towards a better understanding of the corresponding behavioral alterations that accompany aging.

## 1. Introduction

### 1.1. Proline Functions in Stress Protection

PYCS (Pyrroline-5-Carboxylate Synthetase) and PYCR (Pyrroline-5-Carboxylate Reductase) are two key enzymes that control the formation of L-proline from L-glutamate [1,2]. The elevation of exogenous proline level, by either adding exogenous proline molecules or by introducing proline synthesis enzymes to elevate the endogenous proline level significantly protected cells/organisms from environmental stress [3,4]. Although the mechanism was still unclear, scientists have proposed the exogenous proline function as either an osmolyte to reduce the osmotic stress, a chemical chaperone to prevent protein from aggregation, a metal chelator to chelate the toxic metals, and an ROS (reactive oxygen species) scavenger to reduce the ROS stress, maintain the proper NADP+/NADPH level in the cytosol, or increase total GSH (Glutathione) to protect the intracellular reduced GSH [5]. On the other hand, if the proline content was reduced below the essential level, it was conceivable that premature aging might happen in humans, due to a genetic deficiency. Evidence has shown that patients carrying either homozygotic or heterozygotic mutations on the *PYCR1* gene displayed typical premature aging symptoms of lax wrinkled skin, osteopenia, joint hyper laxity, and postnatal growth delay [6,7,8,9,10].

### 1.2. Animal Model for Human Aging

Several animal models, such as worm [11,12], fly [13,14,15], fish [16,17], and rodent [18,19,20,21], have been utilized to study aging-associated gene functions in vivo. In invertebrates, the short life span makes it easy to perform high-throughput screening of genes that are related to longevity. However, it was difficult to correlate the disease phenotypes in invertebrates with that of human due to differences in anatomy and physiology. So far, the most popular animal model for studying aging was the rodent. Our knowledge on aging associated genes has been widely extended in the past two decades thanks to the rapid advancement of the genome manipulating technology. However, screening anti-aging drugs in the rodent was costly and inefficient due to the constraints of long life-span and high cost for maintenance. 

The fish model was suitable for studying aging-associated phenotypes and the underlying mechanisms for number of reasons. First, several biomarkers, such as lipofuscin, SA-β-gal, and permeability on Smurf dye, have been validated to be associated with senescence or aging [22,23,24,25]. Second, despite that the regeneration ability may impede the research on aging in zebrafish, studies demonstrated that the decline of physiological conditions reflected the aging process [26,27]. Third, in some species, such as zebrafish and medaka, the whole genome has been decoded [28,29,30], and molecular tools for manipulating and editing the fish genome were well developed [31,32,33,34,35,36,37]. Finally, several genetic mutants or transgenic models displaying accelerated aging phenotypes have been created [22,38,39]. All these traits make the zebrafish possible to study the biological functions of specific aging-associated genes in greater detail.

We established a *pycr1* KO zebrafish model by Transcription activator-like effector nuclease (TALEN)-mediated gene knockout approach to study how *pycr1* affects aging to facilitate to screen potential chemicals that restore premature aging phenotypes. We observed that the *pycr1* KO fish exhibit premature aging phenotypes, such as dwarfism, slow swimming ability, loss of fertility at the age of six months, and increased mortality when compared to the wild type fish at same age. In addition, we also identified several behavioral alterations in *pycr1* KO zebrafish. Therefore, our *pycr1* knockout zebrafish model may have potential to serve as a platform to screen the potential anti-aging therapeutics in the future.

## 2. Results

### 2.1. Ablation of pycr1 Gene in Zebrafish by TALEN

**Figure 1:**
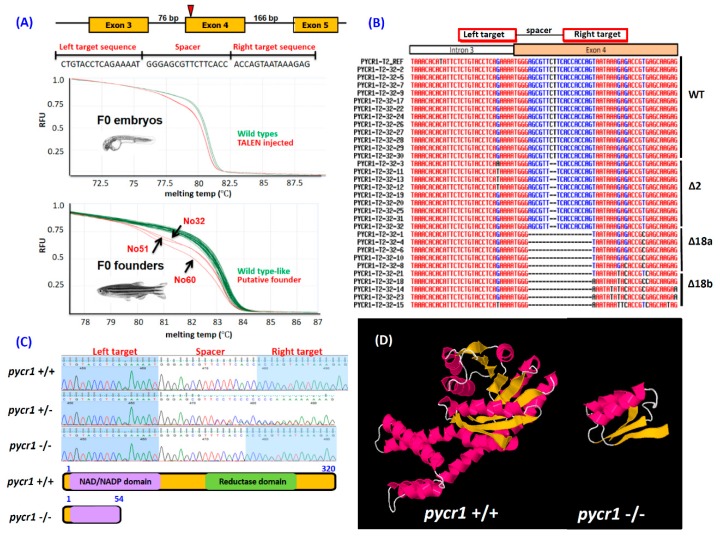
Generation of pyrroline-5-carboxylate reductase 1 (*pycr1)* gene knockout (KO) zebrafish by Transcription activator-like effector nuclease (TALEN) genome editing tool. (**A**) Scheme showed the TALEN left and right arm sequences used to target *pycr1* gene on exon 4 in zebrafish (top panel). The Left and right arm and spacer sequences used for TALEN design were listed in the middle panel. The high-resolution melting assay (HRMA) of F0 fish were showed in the bottom panel. (**B**) Comparison of the Sanger sequencing results of F1 generation that carried potential mutations for *pycr1* gene. (**C**) The stable *pycr1* KO fish used in this study carrying a 2 nucleotide-deletion (delCT) results in a predicted truncate PYCR1 protein with 54 amino acids. (**D**) The predicted three-dimensional structure of PYCR1 protein for WT (+/+) and *pycr1* KO (−/−) fish.

In previous study, morpholino injection has transiently tested the loss-of-function phenotype of the *pycr1* gene, and showed that the decreased expression of *pycr1* induced massive apoptosis in both zebrafish and *Xenopus* embryos [8]. In this study, we established a stable knockout animal to investigate the potential functions of *pycr1* on aging-related processes. In humans, the *PYCR1* gene contained eight exons on chromosome 17, while the *pcyr1* gene in zebrafish contained eight exons on chromosome 3. One TALEN pair targeting zebrafish *pycr1* exon 4 was assembled (Figure 1A, top panel). In vitro transcribed mRNAs were injected into one-cell stage zebrafish embryos. Ten embryos aged at 24 hour-post-fertilization (hpf) were collected, and their genomic DNA was extracted and then screened for potential somatic mutations by high resolution melting assay (HRMA). The results showed the synthesized TALEN mRNAs indeed can target *pycr1* exon 4, and the melting curve of the TALEN mRNA-injected group shifted to the left (red color) when compared with the un-injected group (green color) (Figure 1A, middle and bottom panels). We raised the injected F0 fish to adulthood, and the putative founder fish were screened by HRMA while using the genomic DNA isolated from tail fin clips. Among all of the screened founders, the melting curves of three individuals (No. 32, 51, and 60) showed high shifting to the left (red color), which indicates that there were somatic mutations in the founders (Figure 1A, bottom panel). We outcrossed founder fish No. 32 with wild type (WT) fish and collected single embryos aged at 24 hpf from F1 progeny to perform PCR amplification and Sanger sequencing. Among the 31 F1 progeny that we tested, the mutation rate in F1 progeny was estimated to be 61% (19/31) and three mutation spectrums could be detected, including two base pairs (bp) deletion (9/31), 18 bp deletion pattern 1 (6/31), and 18 bp deletion pattern 2 (4/31) (Figure 1B). The full-length zebrafish *pycr1* gene encodes 320 amino acids with two functional domains, NAD/NADP domain at the N-terminus, and reductase domain at the C-terminus (Figure 1C,D). F1 progeny carrying 2 bp deletion on *pycr1* exon 4 was used to breed homozygotes, which led to premature translational termination of PYCR1 protein at amino acid position 54 (Figure 1C,D). On the contrary, F1 progeny carrying 18 bp deletion (both type 1 and 2), were not used for further analysis, since they only induced the internal deletion of six amino acid residues (Figure 1B).

**Figure A1:**
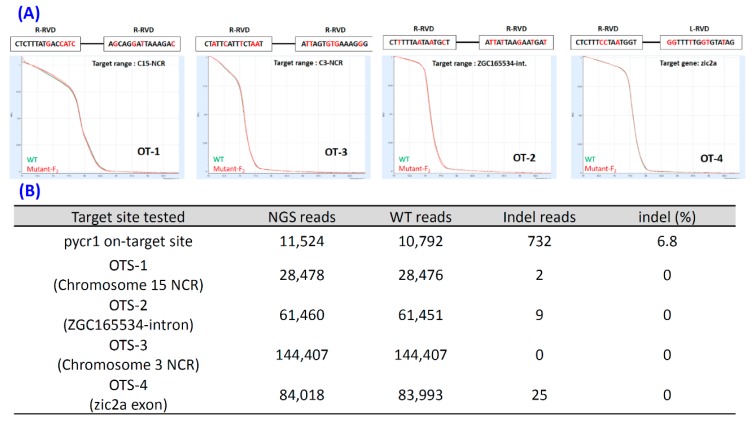
Detection of the potential off-target for *pycr1* gene knockout (KO) by high resolution melting assay and high throughput sequencing. (**A**) Four potential off-target sites (OT-1 to OT-4) were validated by high resolution melting assay. Results showed there are no significant melting curve shifting between WT (green color) and the putative mutants (red color). (**B**) One on-target and four off-target sites were validated by amplicon-based high throughput sequencing. The next generation sequencing (NGS) reads, indel reads and percentage of indels detected in the on-target or off-target sites was listed for comparison.

Next, we investigate whether the *pycr1* TALEN specifically targeted to *pycr1* exon 4. This question was addressed by searching the potential off-target sites within zebrafish genome and examining whether any mutation occurred on the potential off-target sites by HRMA. We identified four potential off-target sites (*C3-NCR*, *C15-NCR*, *ZGC165534*, and *zic2a* genes), which carried high similarity on sequence to the on-target site with 4 to 5 bp mismatch in either the left or the right arm of TALEN pair (Figure A1A). By HRMA analyzing the off target site-specific primers on genomic DNA samples that were isolated from F0 embryos, no shift in the melting curves on four potentials off-target sites was identified, which indicated that the potential off-target effect of *pcyr1* TALEN pair can be ignored (Figure A1A). We also tested the off-target sites by performing amplicon-based next generation sequencing (NGS) on those potential off-targets as well as on-target sites. The results showed indels could only be found at the on-target site, and the somatic mutation rate is estimated around 6.8% (Figure A1B). These results justified that the potential off-target effect of *pycr1* TALEN tested in this experiment can be ignored.

### 2.2. Ablation of pycr1 Gene Induced Senescence and Increased Intestinal Permeability

**Figure 2:**
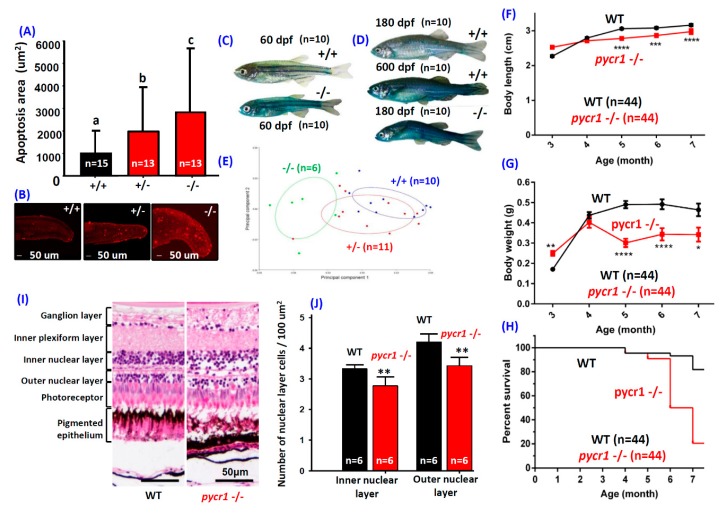
Morphologies of *pycr1* gene knockout (KO) Zebrafish. Quantitative (**A**) and qualitative (**B**) detection of fluorescence apoptotic intensity within tail in the *pycr1* KO fish embryos aged at 24 hour-post-fertilization (hpf) by Terminal deoxynucleotidyl transferase dUTP nick end labeling (TUNEL) staining. Data were presented with mean ± SEM and the significance was tested by one-way ANOVA; *n* = 13–15. The label above column with different letter means reaching significant difference with *p* < 0.05. (**C**) The detection of intestinal integrity in the *pycr1* KO fish at 60 day-post-fertilization (dpf) by Smurf dye staining. (**D**) Detection of intestinal integrity in the WT and *pycr1* KO fish at 180 dpf or natural aging WT fish aged at 600 dpf by Smurf dye staining. (**E**) The Principle Component Analysis (PCA) plot for morphometric analyses among the WT (+/+), heterozygotic (+/−), and homozygotic (−/−) *pycr1* KO fish aged at 180 dpf. (**F**) The growth curves in body length of the WT and the *pycr1* KO fish. (**G**) The growth curves in body weight of the WT and the *pycr1* KO fish. (**H**) The mortality curves of WT and *pycr1* KO fish. (**I**) The retina histological comparison between WT and *pycr1* KO fish aged at 180 dpf. Data were presented with mean ± SEM and the significance was tested by *t*-test in (**F**–**H**), *n* = 44; * *p* < 0.05, ** *p* < 0.01, *** *p* < 0.005, and **** *p* < 0.001. (**J**) The quantitative comparison of retina cell density between WT and *pycr1* KO fish. The data were presented with mean ± SEM and the significance was tested by *t*-test, *n* = 6; ** *p* < 0.01.

**Figure A2:**
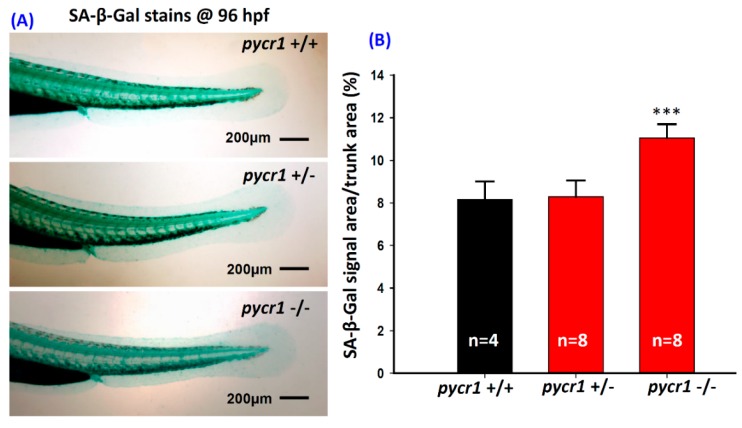
Comparison and quantification of SA-β-Gal staining level in *pycr1* gene knockout (KO) zebrafish embryos. (**A**) Zebrafish embryos aged at 96 hours-post-fertilization (hpf) with either wild type (+/+), *pycr1* heterozygotic (+/−) and homozygotic (−/−) backgrounds were fixed and performed SA-β-Gal staining to evaluate their senescence level. (**B**) Quantitative comparison of the SA-β-Gal staining signal intensity between control and *pycr1* KO fish. The data was expressed as mean ± S.E.M. and the significant difference was tested by one-way ANOVA followed by Tukey multiple comparison analysis. (*** *p* < 0.005).

We demonstrated cell death by Terminal deoxynucleotidyl transferase dUTP nick end labeling (TUNEL) assay and the senescence level by SA-β-Gal assay at the embryonic stage to address how early the aging phenotype can be detected in *pycr1* knockout (KO) fish. The results showed that the degrees of cell death (Figure 2A) and senescence (Figure A2) were significantly elevated in *pcyr1* KO fish at 24 and 96 hpf, respectively. We also detected strong blue stains in two-month-old (Figure 2C) and six-month-old (Figure 2D) *pycr1* KO fish by Smurf Assay. On the contrary, the six-month-old WT fish displayed few Smurf blue staining (Figure 2D). The staining intensity for six-month-old *pycr1* KO fish was comparable to those in natural aging fish aged around two-year-old (Figure 2D). The Smurf Assay (SA) was initially developed in the model organism *Drosophila melanogaster* [40,41] and later validated in multiple other model organisms [42], showing that a dramatic increase of intestinal permeability occurs during aging, so that the Smurf blue dye can enter the fish body and the blue color can be seen on the appearance of fish [25]. This result was consistent with previous study to show induced cell death and senescence in both *pycr1* knock-down zebrafish and *Xenopus* in the morpholino assay [43]. Therefore, we deduced that the loss-of-function of *pycr1* gene induced apoptosis and senescence from the embryonic stage onwards and eventually led to accelerating aging phenotype at adult stages.

### 2.3. Knockout of pycr1 Gene Induces Dwarfism, Aging and Infertility

Zebrafish carrying *pycr1* homozygotic mutation was not lethal, which makes it possible to investigate the adult phenotype. The fecundity in *pycr1* KO fish was greatly reduced when compared with the WT siblings. Due to premature aging, the reproductive performance was greatly reduced in the *pcyr1* KO fish from four months old onwards. The onset of gonadal degeneration was considered to be a reproductive aging process [44]. The *pycr1*-deficient fish can only be successfully bred to produce progeny within the age from three to six months. Mutant fish that were older than six months displayed aging phenotype, including infertile. The gonadal development in both genders of the *pycr1* KO fish was greatly impaired in tissue sectioning, leading to complete infertility at the age older than six months. Morphometrically, the *pycr1* the KO fish was significantly different from those of their WT and heterozygotic siblings in morphometric analysis, where the *pycr1* KO fish showed slim and internally reduced abdomen (Figure 2E). Six-month-old *pycr1* KO fish displayed significantly higher blue staining compared with the control fish in Smurf staining (Figure 2D). We measured the body length, body weight, and survival rate for both the WT and the *pycr1* KO fish. The results showed that the *pycr1* KO fish displayed significantly less body length and body weight, and higher mortality rate from the fifth month onwards (Figure 2F–H).

In addition to dwarfism, we detected strong premature aging phenotype in *pycr1* KO fish and observed more than 80% of *pycr1* KO fish died at the age of seven months (Figure 2H). By histological sectioning, we found that the retina structure of *pycr1* KO fish was severely disorganized. Retinal degenerated process has been considered to be a sign of aging [45,46,47]. Moreover, age-related macular degeneration frequently showed disassociation within the retinal pigment epithelium region [48]. When compared to WT (Figure 2I, left panel), the pigment epithelium layer architecture was disrupted (Figure 2I, right panel) and the cell density of the inner and outer nuclear layers was significantly reduced in the *pycr1* KO fish (Figure 2J). These results suggested that the visual function in *pycr1* KO fish might be altered due to retina degeneration. 

### 2.4. Biological Effects of Premature Aging in Adult pycr1 KO Fish

**Figure 3:**
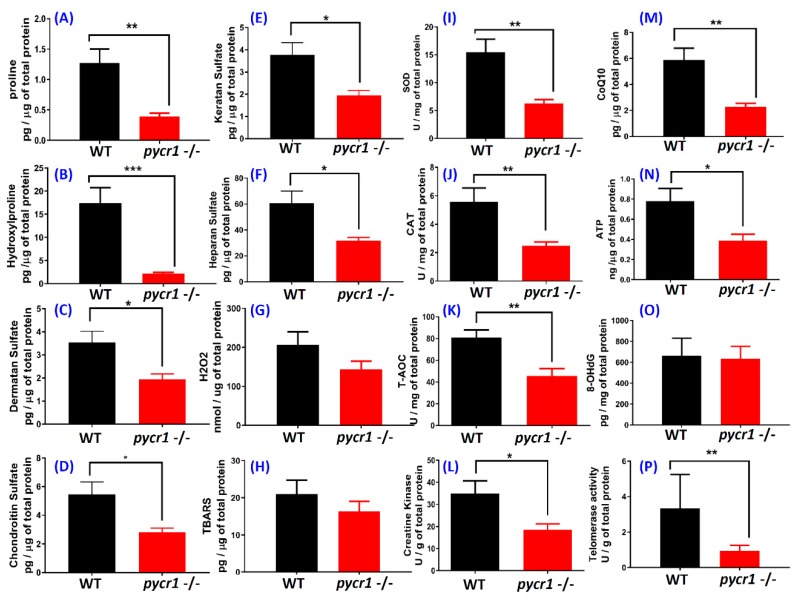
Biochemical analyses in pycr1 gene knockout (KO) zebrafish by Enzyme-linked immunosorbent assay (ELISA). Comparison of proline (**A**), hydroxyproline (**B**), dermatan sulfate (**C**), chondroitin sulfate (**D**), keratan sulfate (**E**), heparan sulfate (**F**), H_2_O_2_ (**G**), thiobarbituric acid reactive substances (TBARS) (**H**), superoxidase dismutase (SOD) (**I**), catalase (CAT) (**J**), total anti-oxidative capacity (T-AOC) (**K**), creatine kinase (**L**), CoQ10 (**M**), ATP (**N**), 8-OHdG (**O**), and telomerase activity (**P**) between wild type (WT) and the *pycr1* KO fish. Data were analyzed by *t*-test, presented with mean ± SEM; *n* = 10; * *p* < 0.05; ** *p* < 0.01; *** *p* < 0.001.

**Figure A3:**
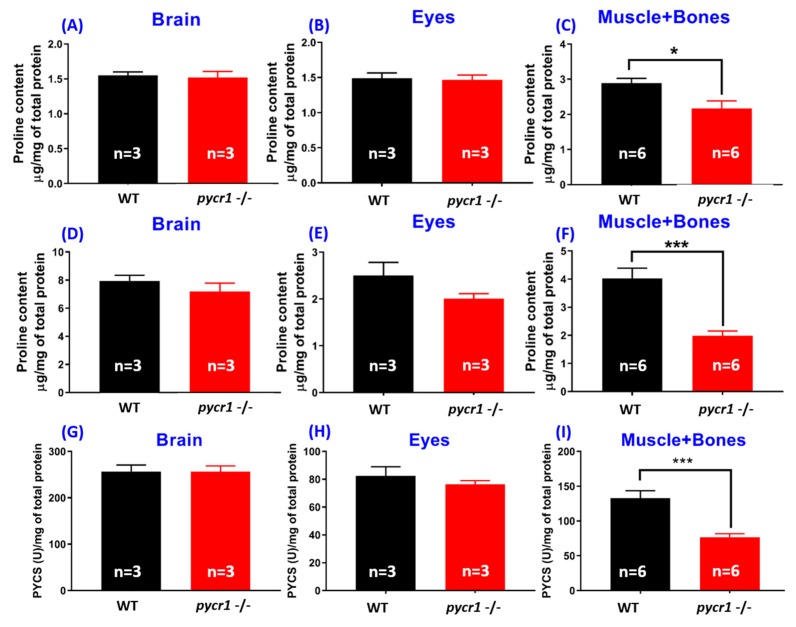
Comparison the proline content and PYCS protein expression levels among diverse tissues between wild type and *pycr1* gene knockout (KO) fish. Five tissues of brain, eyes, skin, bones and muscle were dissected from adult zebrafish aged at six-month-old and subjected to perform biomarkers measurement in Brain (**A**,**D**,**G**), Eyes (**B**,**E**,**H**) and Muscle and Bones mixtures (**C**,**F**,**I**). (**A**–**C**) The proline content was measured by using colorimetric method. The proline (**D**–**F**) and PYCS (**G**–**I**) contents were measured by using ELISA method. The data was expressed as mean ± S.E.M. and the significant difference was tested by *t*-test (sample size *n* = 3–6 and was highlighted in each group, * *p* < 0.05, *** *p* < 0.001).

PYCR enzymes play important roles in proline synthesis. Glutamate can be converted by PYCS enzyme to produce L-P5C intermediate and later catalyzed by PYCR to produce proline. In zebrafish genome, three *pycr* homologous genes, *pycr1*, *pycr2*, and *pycr3*, are located on chromosome 3, 11, and 20, respectively. By enzyme-linked immunosorbent assay (ELISA) with proline-specific antibody, a significantly reduced level of proline in the whole-fish body-extract of the *pycr1* KO fish at adult stage was observed (Figure 3A). Later, we measured proline content among diverse tissues of eye, brain, bone and muscle mixture by either colorimetric or ELISA-based methods. Results demonstrated both measuring methods reach same conclusion, showing that the relative level of proline was significantly reduced in bone and muscle tissue mixtures (Figure A3C,F), while maintained consistent levels in eye and brain tissues (Figure A3A,B,D,E). In addition, the PYCS enzyme also displayed a significant reduction in *pycr1* KO fish’s bone and muscle mixtures (Figure A3I). This result confirms that the *pycr1* gene played an important role on regulating proline biosynthesis and the loss of *pycr1* function would lead to significant reduction on proline production and dysregulate the proline biosynthesis pathway balance. 

Next, we measured the relative amount of extracellular matrix in *pycr1* KO fish, which was suggested to have negative relationship with aging [41,42,43]. By measuring hydroxyproline (a key component for collagen fiber) (Figure 3B) and four glycosaminoglycans (GAGs), including dermatan sulfate (DS) (Figure 3C), chondroitin sulfate (CS) (Figure 3D), keratin sulfate (KS) (Figure 3E), and heparan sulfate (HS) (Figure 3F), we found that their relative amounts significantly declined in the *pycr1* KO fish when compared with WT fish. This result supports that the PYCR1 protein plays an important role in extracellular matrix homeostasis. 

Previous studies showed that PYCR1 is a mitochondrial associated protein, which collaborated with RRM2B to protect cells from oxidative stress. The *pycr1* gene knockdown led cells sensitive to oxidative stress [49]. By either ELISA or enzymatic assays, we found the superoxidase dismutase (SOD) (Figure 3I) and total anti-oxidant capacity (T-AOC) (Figure 3K) were sharply reduced in the *pycr1* KO fish. However, the free radial species H_2_O_2_ (Figure 3G) and lipid peroxidation byproduct of thiobarbituric acid reactive substances (TBARS) showed no difference between the WT and *pycr1* KO fish (Figure 3H). In addition, the creatine kinase activity (Figure 3L), mitochondrial energy producer of coenzyme Q10 (CoQ10) (Figure 3M), and ATP (Figure 3N) levels were greatly reduced in the *pycr1* KO fish. These results are consistent with PYCR1 [44] and proline [4,5] playing important roles in fighting oxidative stress. Moreover, we found the telomerase activity was greatly reduced in the *pycr1* KO fish (Figure 3P). In summary, our data showed that the loss of *pycr1* prompted cells to expose to high stress condition and may play an important role in inducing aging and aging-related phenomenon in the *pycr1* KO fish. 

### 2.5. Knockout of pycr1 Gene Induces pycr1 Transcripts Degradation

**Figure A4:**
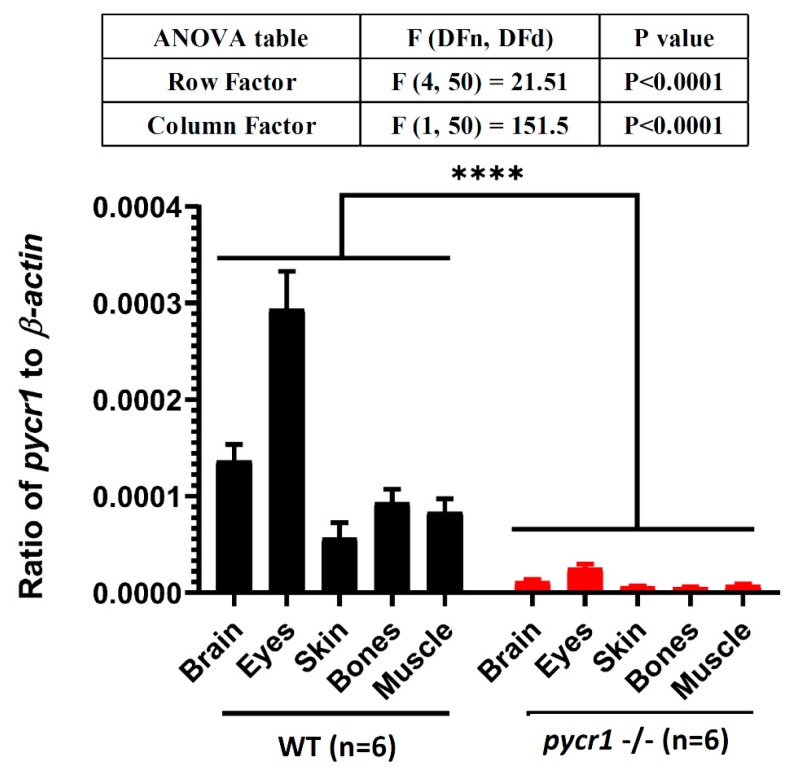
Comparison the *pycr1* mRNA expression level among diverse tissues between wild type (WT) and *pycr1* gene knockout (KO) zebrafish. Five tissues of brain, eyes, skin, bones and muscle were dissected from adult zebrafish aged at six-month-old and subjected to perform real time RT-PCR for mRNA expression quantification. The relative *pycr1* expression level were normalized by using a house keeping *β-actin* gene. The data was expressed as mean ± S.E.M. and the significant difference was tested by two-way ANOVA (sample size *n* = 6 for each tissue samples, **** *p* < 0.0001).

The 2bp deletion mutation that was created by TALEN-mediated gene editing generated the nonsense mutation for pycr1 gene in zebrafish. The nonsense-mediated mRNA decay (MND) effectors are essential in embryonic development and survival [50,51]. Total RNA extracted from diverse tissues, like eye, brain, skin, bone, and muscle were subjected to perform cDNA synthesis. Later, we performed realtime PCR using pycr1-specific primers to test whether the nonsense pycr1 transcripts activate the MND reaction. The results showed the relative mRNA expression levels of pycr1 normalized by β-actin (as an internal control) in the diverse tissue of pycr1 KO fish were significantly lower than those in WT fish by using two-way ANOVA test (Figure A4). Therefore, our data supports that the MND reaction plays a role on degrading the nonsense pycr1 transcripts in pycr1 mutants. 

### 2.6. pycr1 Knockout Fish Showed Multiple Behavioral Alterations

**Figure 4:**
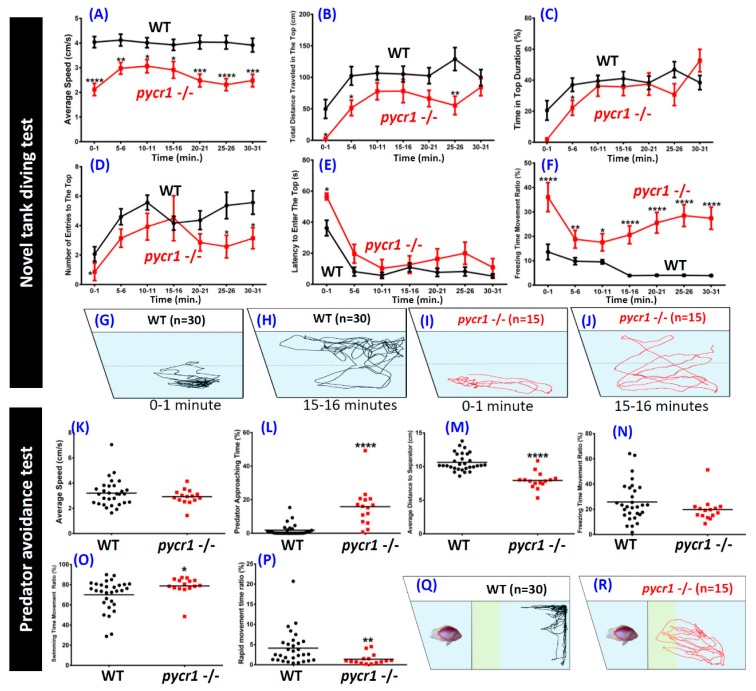
Comparison of behavioral endpoints in novel tank exposure test and predator avoidance test in wild type and *pycr1* gene knockout (KO) zebrafish. (**A**) Average speed, (**B**) total distance traveled in the top area, (**C**) duration time in the top of the tank, (**D**) number of entries to the top, (**E**) latency to entry to the top, (**F**) freezing time to movement ratio, (**G**) and (**H**) locomotion trajectories of wild type (WT) fish before and after acclimation, respectively, (**I**) and (**J**) locomotion trajectories of the *pycr1* KO fish before and after acclimation, respectively. (**K**) Average speed, (**L**) predator approaching time, (**M**) average distance to separator, (**N**) freezing time to movement time ratio, (**O**) swimming time to movement time ratio, (**P**) rapid movement time ratio of WT and *pycr1* KO fish. The data for novel tank test (**A**–**F**) were expressed as the mean ± SEM and analyzed by unpaired *t*-test (WT *n* = 30; *pycr1* KO *n* = 14; * *p* < 0.05; ** *p* < 0.01; *** *p* < 0.001; **** *p* < 0.0001). The data for the predator avoidance test (**K**–**P**) were expressed as the mean and analyzed by Mann–Whitney test (control *n* = 30; *pycr1* KO *n* = 15; * *p* < 0.05; ** *p* < 0.01; **** *p* < 0.0001). (**Q**) and (**R**) locomotion trajectories the of WT and *pycr1* KO fish, respectively.

In addition to intrauterine growth retardation and skeletal abnormality (hip dislocation), mutated *PYCR1* in human patients caused abnormalities in the central nervous system, such as mental retardation, microcephaly, corpus callosum agenesis, and epilepsy [52]. Furthermore, an analysis of the behaviors of the *pycr1* KO fish was conducted. Similar to the rodent open field test, the novel tank assay is a method of evaluating zebrafish anxiety level by exploiting the innate behavior of zebrafish to seek protection in an unfamiliar environment [53]. The adaptation ability, including locomotion activity and exploratory behavior of the *pycr1* KO fish, was demonstrated in a novel tank. When introduced into a novel environment, zebrafish have a natural tendency to spend the majority of time at the bottom and then expand their position of swimming to include the higher portions of the test tank gradually over a period of minutes [54]. The degree of ‘bottom dwelling’ has been interpreted as an index of anxiety in zebrafish [55]. Using this test, some behavioral parameters were able to be collected and compared to assess anxiety. After locomotion activity was quantified, we found the average speed (Figure 4A), total distance traveled in the top portion of the tank (Figure 4B), time in the top portion of the tank duration (Figure 4C), and number of entries to the top (Figure 4D), were significantly reduced in *pycr1* KO fish. The latency to enter the top portion of the tank (Figure 4E) and the freezing time to movement ratio (Figure 4F) of the *pycr*1 KO fish were significantly increased at every time point (Video S1). The locomotion trajectories of the novel tank test before and after acclimation are summarized in Figure 4G–J. Reduced locomotion activity/freezing that observed during this test indicated heightened anxiety [53]. In the rodent open field test, anxiety in rats is also typically measured as a suppression of exploratory behavior, including freezing and a reduction in locomotor activity [56]. These results demonstrated that the *pycr1* KO fish exhibited stronger anxiety-like behavior than the WT fish, and a longer habituation time was needed than for the normal zebrafish when they were exposed to the new environment. 

Secondly, we carried out predator avoidance test to compare the innate predator escape response that is induced by the predator fish convict cichlid (*Archocentrus nigrofasciatus*) between the *pycr1* KO and the WT fish. It was intriguing to find that the *pycr1* KO fish displayed less fear response as triggered by the predator fish, which was represented by significantly more predator approaching time (Figure 4L) and a shorter average distance to the glass separator between the zebrafish and the predator (Figure 4M). While there was no significant difference in the average speed between the control and *pycr1* mutant fish (Figure 4K), the aberrant distribution of the *pycr1* KO fish movement types (freezing movement, swimming movement, and rapid movement) (Figure 4N–P) as compared with the WT fish was identified in this test (Video S2). Figure 4Q,R summarize the locomotion trajectories of the predator avoidance test.

**Figure 5:**
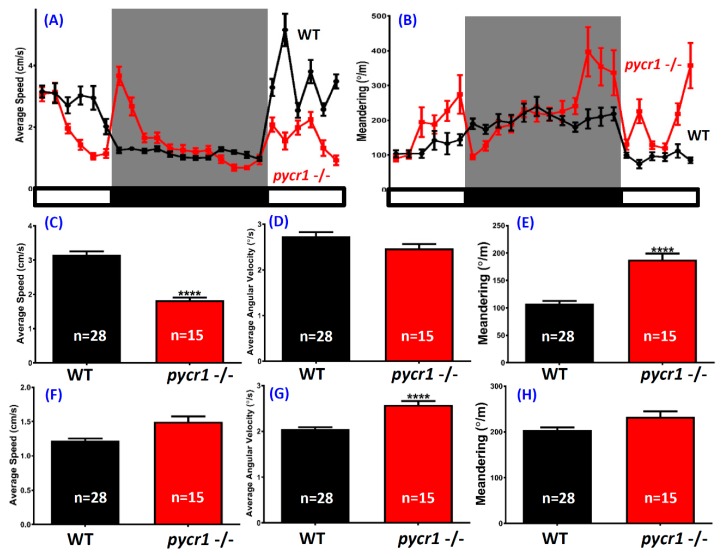
Comparison of untreated wild type (WT) and *pycr1* gene knockout (KO) zebrafish in the circadian rhythm test. (**A**) Circadian patterns of average speed and (**B**) meandering in during light/dark cycles. (**C**) Average speed, (**D**) average angular velocity, and (**E**) meandering during the light cycle. (**F**) Average speed, (**G**) average angular velocity, and (**H**) meandering during the dark cycle. The data are expressed as the mean ± SEM and analyzed by Mann–Whitney test (control *n* = 28; *pycr1* KO *n* = 15; **** *p* < 0.0001).

Age-related changes in circadian rhythmicity have become an important area of research, since cognitive dysfunctions in human dementias were often associated with circadian rhythm and sleep disorders [57]. For the circadian cycle test, a custom designed infrared light box was used to detect and compare the locomotion activity of the *pycr1* mutant fish during the light and dark cycles. We discovered that the *pycr1* KO fish displayed a dysregulated circadian rhythm phenotype. In the light cycle, less average speed (Figure 5C) and higher meandering of the mutant fish (Figure 5E) indicated the irregular movement and reduced locomotion activity. In addition, average angular velocity in the light cycle was also lower, even though it was not significant (Figure 5D). This result is in consistent with our novel tank test result, which showed a hypoactivity behavior in mutant fish that may indicate anxiety. Meanwhile, the total locomotion activity of the mutant fish was not significantly different when compared to the control in the dark cycle, as well as the slightly higher level of the average speed (Figure 5F) and meandering of the *pycr*1 KO fish (Figure 5H). The only difference was presented in the average angular velocity endpoint, which was significantly higher in the *pycr1* KO fish (Figure 5G). However, an irregular pattern of mutant fish activity was detected during the dark cycle, which was illustrated by high average speed on the first two time intervals of dark cycle, continued with constant level of average speed for the following six time intervals, and it gradually decreased until the transition from the light cycle to dark cycle (Figure 5A,B). To sum up, the *pycr1* mutant fish locomotion activity pattern was different with the control fish in both of the day and night cycles, and this phenomenon might be caused by anxiety that was exhibited by the mutant fish and/or circadian rhythm defects. There is also possibility that this dysregulation related to the low level of melatonin, a hormone that is produced by the pineal gland at night to serve as a time cue to the biological clock that was detected in the current experiment (Table 1). Nevertheless, this result showed us that *pycr1* mutant fish had sleep dysregulation. 

**Figure 6:**
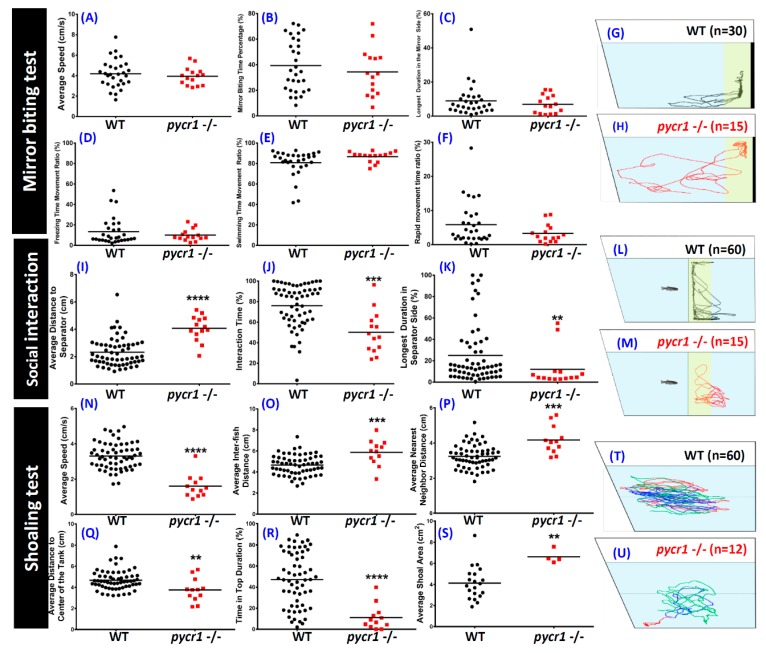
Comparison of behavioral endpoints of untreated wild type (WT) and pycr1 gene knockout (KO) zebrafish in mirror biting (**A**–**H**), social interaction (**I–M**) and shoaling test (**N**–**U**). (**A**) Average speed, (**B**) mirror biting time percentage, (**C**) longest duration in the mirror side, (**D**) freezing time movement ratio, (**E**) swimming time movement ratio and (**F**) rapid movement time ratio for mirror biting assay. The mirror was positioned in the right side. The mirror approaching zones are highlighted in yellow color. Locomotion trajectory of wild type (**G**) and pycr1 KO (**H**) zebrafish in mirror biting test. (**I**) Average distance to the separator, (**J**) interaction time percentage (the fraction of time when the test fish stay in the yellow-highlighted zone), (**K**) longest time stay in the separator side during social interaction test. Locomotion trajectory of the wild type (**L**), and the pycr1 KO (**M**) zebrafish in the social interaction test. The normal wild type zebrafish is placed in the left side and test KO fish is placed in the right site with a transparent glass plate inserted in between to separate both test animals. The social interaction approaching zones are highlighted in yellow color. (**N**) average speed, (**O**) average inter-fish distance, (**P**) average nearest neighboring distance, (**Q**) average distance to the center of the tank, (**R**) time in the top duration, (**S**) average shoal area for the shoaling test (shoaling size *n*= 3). Locomotion trajectory of the wild type (**T**) and the pycr1 KO (**U**) zebrafish in the shoaling test. The shoaling trajectories for each single fish were labeled in different color. The data for mirror biting test were expressed as the mean and analyzed by Mann-Whitney test (control *n* = 30; *pycr1* KO *n* = 15; ** *p* < 0.01; *** *p* < 0.001; **** *p* < 0.0001). The data for social interaction test were expressed as the mean and analyzed by the Mann–Whitney test (control *n* = 60; *pycr1* KO *n* = 14; ** *p* < 0.01; *** *p* < 0.001; **** *p* < 0.0001). The data for shoaling test were expressed as the mean and analyzed by Mann–Whitney test (*n* control *n* = 60; *pycr1* KO *n* = 12; ** *p* < 0.01; *** *p* < 0.001; **** *p* < 0.0001).

To evaluate the social behavior of fish, we conducted a mirror biting assay to test the aggressiveness of the fish. Generally, zebrafish social/aggressive behavior, the mirror image stimulation, is a well-established fish paradigm. Zebrafish display boldness by biting or butting the mirror when placed in a tank with conspecific, which is highly relevant to social behavior [50]. There was no significant difference for the aggressive behavior between the *pycr1* KO and the WT fish when comparing the duration time that zebrafish stayed along the mirror side. The average speed (Figure 6A), mirror biting time percentage (Figure 6B), longest duration in the mirror side (Figure 6C), and all fish movement types (Figure 6D–F) (Video S3) were measured. The locomotion trajectories of the mirror biting test are summarized in Figure 6G,H.

Furthermore, social interaction and shoaling test were demonstrated. Based on a similar rodent paradigm, the social interaction test is another useful model to study social phenotypes. In this test, we assessed zebrafish sociability by observing their interactions with the conspecifics [58]. Even though this test and mirror biting test present comparable visual cues to the animals, there is a major difference between these two tests. In the mirror biting test, zebrafish reflection in the mirror that was placed in a tank become its stimulus. Meanwhile, in the social interaction test, the stimulus is its conspecific. These two stimuli have their own specific characteristics in how they stimulate the test fish behavior response. Since the mirror biting test stimulus is a test fish reflection, it acts same as the test fish. However, social interaction test stimulus behaves different with mirror biting test stimulus. In the social interaction test, the occurrence of distinct behavior of the stimulus and the test fish is possible, as the stimulus is also a real fish. This difference might become one of the reasons why the mirror biting test is traditionally used for studying zebrafish aggressive behavior, while the social interaction test is used to assess zebrafish sociability by observing the interaction between several fish [58]. 

Shoaling, which is another social behavior, represents the interaction of a group of animals moving together in coordinated movements, which is an important evolutionarily conserved behavior. In zebrafish, shoaling is an innate behavior that is maintained at a relatively high and stable level throughout lifespan. Previously, shoaling assays in zebrafish have been applied to study ontogenesis, behavioral organization, genetic factors, effects of environmental stressors, and pharmacological modulation [59]. The social interaction test results showed that *pycr1* KO fish were not interested in their conspecifics and displayed distinctive social withdrawal phenotype, which were supported by a longer average distance to the separator (Figure 6I), less interaction time (Figure 6J) and less the longest duration in the social interaction zone (the yellow-highlighted zone) (Figure 6K) when compared with the WT (Video S4). Figure 6L,M display the locomotion trajectories of fish for the social interaction test.

Meanwhile, based on the shoaling test, most of the *pycr1* KO fish could not form a shoal when they were swimming together, which was supposed to be due to a decrease of the average speed (Figure 6N), leading to a deformation of shoaling and an increase of the average inter-fish distance (Figure 6O), the average nearest neighbor distance (Figure 6P), and a broader shoal area (Figure 6S). The *pycr1* KO fish displayed less exploratory behavior, which was indicated by a shorter average distance to the center of the tank (Figure 6Q) and less time in the top portion of the tank duration (Figure 6R) (Video S5). Figure 6T,U summarize the locomotion trajectories of the shoaling test. Despite the correlation between behavioral abnormalities and aging, by conducting multiple-endpoint behavioral tests, it is pioneering for us to report that *pycr1* KO fish displayed multiple behavioral abnormalities.

### 2.7. Pycr1 KO Fish Display Color Preference Abnormality

**Figure 7:**
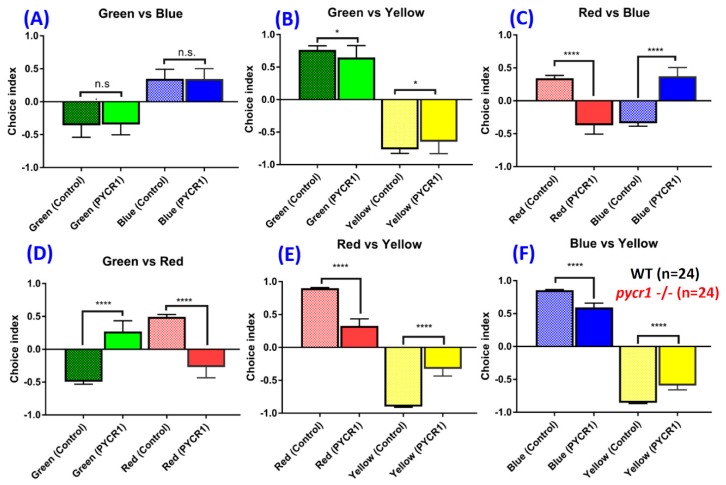
The effect of the *pycr1* gene knockout (KO) on color preference performance. (**A**) Green and blue combination, (**B**) green and yellow combination, (**C**) red and blue combination, (**D**) green and red combination, (**E**) red and yellow combination, and (**F**) blue and yellow combination. The data were presented with mean ± SEM and analyzed by one-way ANOVA, followed by Tukey multiple comparison analysis with (*n* = 24; ns = non-significant; * *p* < 0.05; ** *p* < 0.01; *** *p* < 0.001; **** *p* < 0.0001).

In the histological assay, we found that the retina structure of *pycr1* KO fish was severely disorganized, including disrupted architecture of the pigment epithelium layer (Figure 2G). In addition, reduced cell density in the inner and the outer nuclear layers was observed (Figure 2G,H). Therefore, performing color preference test investigated whether the *pycr1* KO fish exhibited any color perception alteration. We found that the color preference of the WT from most to least was red, blue, green, and yellow. However, in *pycr1* KO fish, the color preference was blue, green, red, and yellow instead (Figure 7). The color preference for the WT and the *pycr1* KO fish always showed significant difference in red color partition, which suggests that the *pycr1* mutants may cause alterations that are related to red and yellow color vision, as shown in red/blue combination (F_3,64_ = 9.204, *p* < 0.0001) (Figure 7C), green/red combination (F_3,64_ = 9.144, *p* < 0.0001) (Figure 7D), red/yellow combination (F_3,64_ = 11.78, *p* < 0.0001) (Figure 7E), green/yellow (F_3,64_ = 3,926, *p* = 0.0433) (Figure 7B), and blue/yellow combination (F_3,64_ = 8.447, *p* < 0.0001) (Figure 7F).

### 2.8. Pycr1 KO Fish Display Normal Short-Term Memory

**Figure A5:**
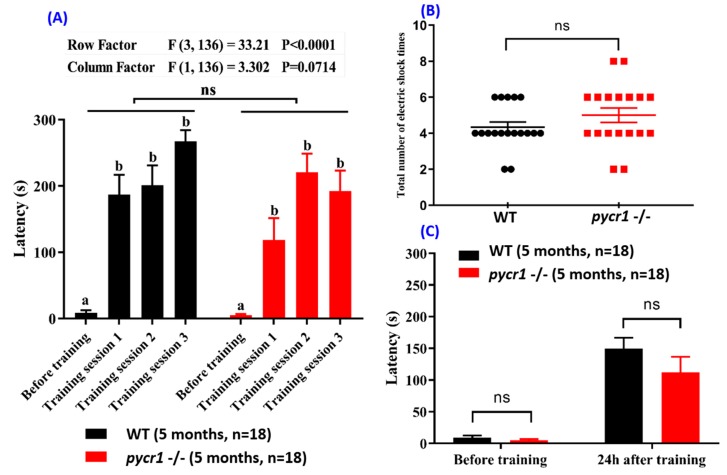
Comparison of the short-term memory between wild type (WT) and *pycr1* gene knockout (KO) zebrafish by passive avoidance test. (**A**) Comparison of the learning latency between control (black color) and *pycr1* KO fish (red color) moving into the black chamber coupled with electric shock. (**B**) Comparison of the total electric shock number given for training between control (black color) and *pycr1* KO fish (red color). (**C**) Comparison of the memory latency between control (black color) and *pycr1* KO fish (red color) moving into the black chamber coupled with electric shock. The data was expressed as mean ± S.E.M. and the significant difference was tested by two-way ANOVA in (**A**) and (**C**), and by *t*-test in (**B**). The fish age (5-month-old) and number (*n* = 18) used in this assay were also highlighted. ns, no significance.

Since PYCR1 deficiency in human caused mild mental retradation [60], it is intriguing for us to explore whether the pycr1 KO fish display dementia phenotype. To archive this goal, we performed passive avoidance test to compare the short-term memory between WT and pcyr1 KO zebrafish that were aged at five month-old. Usually, zebrafish can be trained to build up the short-term memory after three eletric shock trainings were given in the passive avoidance test (Figure A5A). The results showed there are no differences on either learning latency (Figure A5A), total electric shock number given for training (Figure A5B), or memory latency (Figure A5C) between control and pycr1 KO fish. Therefore, we concluded that zebrafish carrying pycr1 gene deficiency display normal short-term memory as their WT counterparts.

### 2.9. Detection of Neurotransmitter Expression in pycr1 KO Fish Brain and Other Tissues

The behavioral abnormality in the *pycr1* KO animal has not yet been reported in other model animals. We measured different neurotransmitter levels in the fish body and brain to understand the mechanism. By ELISA assays with target-specific antibodies for neurotransmitters, including GABA, dopamine, serotonin, melatonin, epinephrine, norepinephrine, acetylcholine, etc., we found that all of the neurotransmitter levels displayed no significant difference in brain tissues between WT and *pycr1* KO fish (Table 1). This result demonstrated that *pycr1* loss-of-function may not cause the dysregulation of brain neurotransmitters. Nonetheless, when ELISA measured the neurotransmitter levels in the other body parts, the results showed most of the neurotransmitters including dopamine, GABA, serotonin, melatonin, norepinephrine, epinephrine, acetylcholine, glycine, and glutamate were strongly downregulated in the whole-body extracts (except in the brain tissues) in the *pycr1* KO when compared to the WT fish (Table 1). Therefore, we proposed that the behavioral alteration in *pycr1* KO fish might be the secondary response due to imbalance homeostasis of neurotransmitters in the peripheral tissues. The brain tissue, on the contrary, still maintains constant neurotransmitter level when the *pycr1* gene function was compromised.

## 3. Discussion

### 3.1. Comparison of pycr1 Knockdown and Knockout Phenotypes

The PYCR1 protein is a key enzyme controlling proline synthesis and it is associated with progeria disease in humans when its function is compromised. Patients carrying *PYCR1* gene deficiency showed proline metabolic disorder, which leads to premature aging phenotypes, intrauterine growth retardation, triangular facial gestalt, psychomotor retardation, hypotonia, and ophthalmologic abnormalities, and progeroid cutaneous manifestations [61]. In previous studies, transient morpholino-based knockdown in zebrafish and *Xenopus* embryos showed developmental retardation and high level of apoptosis when the *pycr1* gene activity was compromised [49]. In this study, we generated a stable zebrafish mutant line carrying *pycr1* gene deficiency by the TALEN-mediated genome editing tool, and found a strong elevation of apoptosis and senescence levels in *pycr1* KO fish. In addition to elevated apoptosis and senescence levels, adult *pycr1* KO fish at two-months old already displayed aging symptoms that are based on strong Smurf dye staining, showing their intestinal cell permeability to be already compromised (Figure 2C). Biochemical assays showed that *pycr1* KO fish have a significant reduction in anti-oxidative capacity, which supports that the PYCR1 protein serves as a stress scavenger to remove the ROS induced in a stressed environment. In addition, we also observed that the *pycr1* KO fish displayed a low energy level in their tissues with reduced creatine kinase activity, ATP, and CoQ10 levels, which might be corelated with mitochondrial dysfunction. Therefore, when compared to previous transient knockdown experiment [49], we explored more interesting aging related phenotype in *pycr1* KO zebrafish. This zebrafish mutant carrying premature aging phenotype provides the research community an excellent in vivo model for aging related studies and for anti-aging drug screening in the future.

### 3.2. Comparison of pycr1 KO Fish with Other Aging Fish Models

Aging is considered to be a complicated process that is controlled by genetic and environmental factors. Among several aging-associated mutant models [62], the telomerase-deficient zebrafish showed phenotypes similar to the *pycr1* KO fish, with an increase in apoptosis, infertility, and retina regeneration [63]. The expression of telomerase is highly associated with telomere length and the efficiency of tissue regeneration [64]. On the other hand, p53 activation was also respond to telomere dysfunction, which led to aging [65]. In this study, the telomerase activity in the *pycr1* KO fish was significantly reduced (Figure 3P). However, the marker for DNA damage (8-OHdG) was found to have no significant difference between the *pycr1* KO and the WT fish (Figure 3O), which suggests that the aging mechanism of the *pycr1* KO fish was different from that of the *tert*−/− mutant fish. Further studies are needed to elucidate the potential mechanism on p53 activation and telomere dysfunction in *pycr1* KO fish. 

In addition, according to our results, the aging phenotypes in the *pycr1* KO fish might come from mitochondria degeneration and extra cellular matrix (ECM) aberration. Generally, mitochondrial dysfunction has been classified as one hallmark of aging. The PYCR1 protein has been reported to be essential in cell proliferation and maintaining the structural integrity of mitochondria [49]. Therefore, we proposed that the low energy levels in *pycr1* KO fish could be caused by the degenerated mitochondria due to a loss of integrity. This energy deficiency might be insufficient to maintain normal cell function, such as transcription and translation control. This might be the reason that explains why most of the biomarkers we measured were downregulated in *pycr1* KO fish.

### 3.3. Possible Mechanism for Behavioral Alteration in pycr1 KO Fish

In this study, we proposed a novel finding that the *pycr1* KO zebrafish model displayed multiple behavioral changes that are associated with aging. However, no significant change of neurotransmitter levels was detected in the brain tissues of mutant. Instead, we observed the lower level of neurotransmitters in fish body extract of mutants when compared with wild type fish (Table 1). Collagen that is made of large amount of proline and hydroxyl-proline accounts for 1/3 of animal bodies and it is the major component for connective tissues, such as bones, muscles, and blood vessels [66]. Therefore, the *pycr1* KO fish were short of proline synthetase and unable to synthesize enough proline or hydroxyl-proline derived from proline could not develop enough connective tissues to support normal physical activity, leading to poor locomotion ability and aberrant behaviors. The brain tissue does not contain a high percentage of proline when compared with the peripheral tissues in fish, so the brain’s function might be less affected during the depletion of proline. However, the peripheral tissues were enormously affected and the neurotransmitter amounts significantly decreased in the body of *pycr1* KO fish when compared to that of wild type fish. Proline, in addition to serving as basic amino acid component, itself also plays a role as free radical scavenger. Evidences that were collected from either in vitro or in vivo experiments demonstrated that the cell viability and tolerance can be improved when the proline level is elevated [4,67]. In our *pycr1* KO fish, we found that the tissue proline content display sharply declined in bone and muscle. Brain and eye tissues, on the contrary, displayed relatively low and constant proline levels after the *pycr1* gene function is compromised. This might be the reason why the neurotransmitter level keeps constant in the brain, while displaying great alteration in peripheral tissues in *pycr1* KO fish. Furthermore, the neurotransmitters immediately diffused into blood after secretion, and the secretion was limited to a certain region that was not separated from the whole brain. This might be the reason leading to a dilution of neurotransmitters, so ELISA in the brain tissues could not distinguish the concentration difference.

The low level of serotonin, norepinephrine, and melatonin in the body extracts might be related to less aggressive, less social interaction, less fear to predator, and dysregulated circadian rhythm in *pycr1* KO fish. Moreover, according to the color preferences in *pycr1* KO fish, we hypothesized that the color preference alteration might be correlated with either degenerated retina, as evidenced in retina histology (Figure 2G) or depression due serotonin downregulation. The color preference index got reversed changes specific in red-blue and red-green combinations, which suggests that the cone cells responsible for receiving long-wavelength light (especially for red color) might lose their function. However, further studies, such as in situ hybridization, RT-PCR, or immunohistochemistry with cone-specific probes will be needed to clarify whether the color preference alteration is corelated to cone-specific marker gene expression alteration in *pycr1* KO fish.

**Figure 8:**
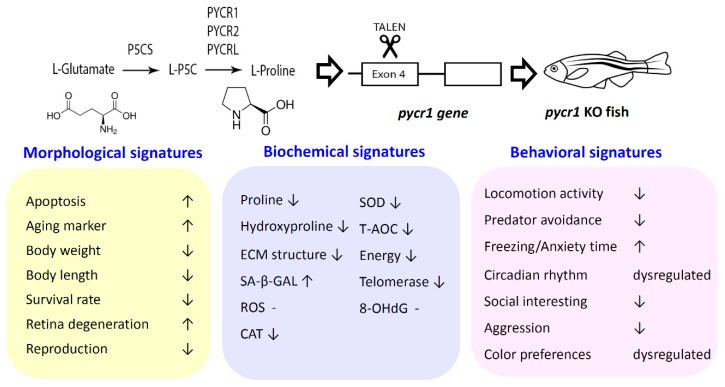
Schematic representation of the morphological, biochemical and behavioral signatures detected in *pycr1* gene knockout (KO) zebrafish. The *pycr1* gene function and the method used to knockout zebrafish *pycr1* gene were listed in the upper panel. The signatures at morphological (yellow color), biochemical (blue color), and behavioral (pink color) levels are summarized in the bottom panel.

In conclusion, in this study, we created a zebrafish aging model carrying *pycr1* gene deficiency for the first time. We provided morphological, cellular, molecular, and behavioral evidences to show how this *pycr1* KO fish displays aging and behavioral alteration phenotypes. We also explored its potential underling mechanism by assessing biomarker and neurotransmitter expression. This *pycr1* KO zebrafish model provided a novel and precious in vivo platform for examining the PYCR1 functions in the aging process (summarized in Figure 8). It provided a good animal model to study the premature aging disease (autosomal recessive cutis laxa, ARCL) that is caused by *pycr1* deficiency in humans and it might be applied as an in vivo platform for discovering drugs for the treatment of premature human aging disorders in the future.

## 4. Experimental Procedures

### 4.1. Animal Ethics and Rearing

The Committee for Animal Experimentation of the Chung Yuan Christian University approved all of the experimental protocols and procedures involving zebrafish (Number: CYCU10107, issue date 23 August 2012). All of the experiments were performed in accordance with the guidelines for laboratory animals. Wild-type AB strain and *pycr1* KO zebrafish were maintained in a recirculating aquatic system at 28.5 °C with a 10/14-h dark/light cycle according to standards. Circulating water in the aquarium was filtered by reverse osmosis (pH 7.0–7.5). The zebrafish were fed twice a day with lab-grown brine shrimp. For behavioral tests, we used adult zebrafish aged around 6–10 months.

### 4.2. Body Length, Body Weight and Survival Rate Measurement

Mixed gender of WT, *pycr1* +/− and *pycr* −/− zebrafish were separately raised in 10L tanks until they reached three months old (n=60 for each group). From three months onwards, WT (*pycr1* +/+), *pycr1* +/− and *pycr1* −/− zebrafish were transferred and subsequently raised in 100L tanks. All of the fish were fed with artemia twice per day and kept at constant temperature of 28°C. The standard body length, body weight, and survival rate were measured every month when fish were aged from three months onwards until seven months.

### 4.3. Histology

For histology, the tissues were dissected and embedded in Technovit 7100 resin (Heraeus Kulzer) or 4% Paraformaldehyde (PFA). The samples were sectioned at 5 μm interval and counter-stained with H&E (Merck) to visualize the nuclear position according to previously described protocol [68]. For the paraffin section, adult zebrafish were fixed in 4% paraformaldehyde/PBS for one day and transferred to Davidson’s solution (30% Ethyl alcohol (95%), 10% acetic acid, 20% formalin, and 30% double distill water) for three days at room temperature and were later subjected to paraffin sectioning and immunohistochemistry, according to the protocols described previously [68].

### 4.4. Morphometric Analysis

The image files were first converted to .tps format using TpsUtil for morphometric analysis (http://life.bio.sunysb.edu/morph/soft-utility.html). We digitized the image landmark using TpsDig2 tool. Later, we perofrmed procrustes analysis for zebrafish using MorphoJ software (http://www.flywings.org.uk/morphoj_page.htm). This MorphoJ software can generate a covariance matrix and then Principal Component Analysis (PCA) can be performed to compare the morphometric difference between the wild type and pycr1 mutant. The dtail protocol for morphometric analysis can be found in supplemetary protocol.

### 4.5. Production of TALEN mRNA

TALEN vectors were purchased from a commercial company (Zgenebio Co, Taipei, Taiwan). The TALEN vectors were linearized with Not1 restriction enzyme and transcribed using mMACHINE SP6 kit (Life Technologies, Carlsbad, CA, USA) to synthesize 5′capping TALEN mRNAs. Following the completion of transcription, poly(A) tailing reaction and DNase I treatment were performed according to the manufacturer’s instructions for the TALEN mRNAs. The in vitro transcribed TALEN mRNAs were filtered and enriched by YM30 column to increase the purity of the TALEN mRNAs.

### 4.6. Microinjection of Zebrafish Embryos

The TALEN mRNAs were co-injected with Transposase mRNA into one-cell stage zebrafish embryos. Each embryo was injected with approximately 2 nl of solution containing 50 pg/nl of TALEN mRNA. On the next day, the injected embryos were inspected under a stereoscope. Only embryos that developed normally were used for further analyses. Genomic DNA was extracted from embryos one day-after injection of TALEN mRNAs.

### 4.7. Identification of Indel and Targeted Mutations by HRMA

Genomic DNA was extracted from the pools of 10 controls or injected embryos. Targeted genomic loci were amplified using primers that were designed to anneal approximately 150 to 200 base pairs upstream and downstream, respectively, from the expected FokI cut site and KOD FX high-fidelity DNA polymerase (Toyobo, Osaka, Japan), according to the manufacturer’s instructions. All of the primers used in this study have been described previously [39]. HRMA was performed by MyGO Pro (IT-IS Life Science Ltd., Mahon, Republic of Ireland). Each target locus was amplified by PCR from the pooled genomic DNA of ten injected embryos. The resulting PCR products were cloned into the pGEM-T vector (Promega) and transformed into *E. coli* bacteria DH5α strain. Thus, each colony represented one PCR amplified sequence. To determine the somatic mutation rates, the National Taiwan University Hospital DNA Sequencing Core sequenced the plasmids DNA isolated from single colonies. The indel mutation rates were determined by the number of colonies containing mutant sequences divided by the total number of colonies sequenced.

### 4.8. Production of pycr1 KO Fish

The potential founders were crossed with the wild-type zebrafish. Three to four days post-fertilization, 20 progenies were pooled and lysed in 25 microliters of the alkaline lysis buffer (25 mM NaOH, 0.2 mM EDTA) and heated at 95 °C for 30 min. Subsequently, the DNA solution was neutralized using 25 microliters of the neutralization buffer (40 mM Tris-HCl, pH8.0). The samples were spun at 13,000 g for five minutes to remove the debris, and the supernatant contained extracted genomic DNA. In general, 20 embryos from each potential founder were screened for the presence of indel mutations by HRMA amplifying the region that surrounds the relevant TALEN FokI cleavage site using a forward primer and a reverse primer. For sequence confirmation, genomic DNA from single embryos was amplified using targeted loci-specific primers. The PCR products were then submitted for Sanger sequencing.

### 4.9. TUNEL Assay

For the TUNEL assay, embryos that were aged 24-h post-fertilization (hpf) were pre-treated with PTU and then processed to 4% PFA fixation for at least 6 h at 48 hpf. Following washing in PBST for 10 min, embryos were stored in 100% methanol at −20 °C for 2 h or longer. Embryos were subsequently incubated with 0.1 M sodium citrate for 30 min. at 70 °C to enhance embryo permeability. Thereafter, the embryos were rinsed twice with PBS and incubated with 5 μL of enzyme solution plus 45 μL of label solution at 37 °C for 1hr following the manufacturer’s instructions (Roche, In Situ Cell Death Detection Kit, TMR red, 12156792910). Finally, the embryos were washed three times in PBS and ready for detecting of red fluorescent signals for cell death.

### 4.10. SA-β-gal Assay

Zebrafish adults and embryos were fixed in 4% paraformaldehyde in phosphate buffered saline (PBS) at 4 °C (for three days for adults and overnight for embryos), and then washed three times for 1 h in PBS (pH 7.4) and once for 1 h in PBS (pH 6.0) at 4 °C. Staining was performed overnight at 37 °C in the staining solution (5 mM potassium ferrocyanide, 5 mM potassium ferricyanide, 2 mM MgCl2, and 1 mg/ml X-gal in PBS adjusted to pH 6.0). All of the animals were photographed under the same conditions using transmitted light under a dissecting microscope. SA-β-gal activity in each animal was quantitated using a selection tool in Adobe Photoshop, following the method described previously. For analyses of embryos, the SA-β-gal-positive areas in the tail fin were counted and normalized to the total tail fin size.

### 4.11. Smurf Dye Staining

Adult zebrafish were immersed in working concentration of Smurf dye (0.003125% working concentration dissolved in fish water, 861146 Sigma) for 30 min. and then rinsed in fish water for another 30 min. for de-staining, according to previous published protocol [25]. In aging zebrafish, darker staining can be detected, due to the increased permeability in the intestinal tissues.

### 4.12. Behavioral Assessment

The novel tank, mirror biting, predator avoidance, social interaction, and shoaling tests were conducted by a special designed zebrafish tower according to our previous published protocol [69]. A special designed chamber equipped with infrared camera by following the method described in previous publication conducted the circadian rhythm [70]. The color preference test was conducted by a special designed device by following the method described in previous publication [71]. The short-term memory was tested by using the passive avoidance method described in our previous published paper [70].

### 4.13. Preparation of Brain and Body Extracts

After the behavioral analysis, the fish were treated in ice water to death. Subsequently, the brain and the fish body were separated and used for homogenate preparation. The tissues were minced and homogenized at medium speed with a Bullet blender (Next Advance, Inc., Troy, NY, USA) with 10 volumes of (v/w) ice cold phosphate saline buffer adjusted to pH 7.2. The samples were incubated on ice for 30 min. and further centrifuged at 13,000× *g* or 15 min. and the crude homogenates were stored in 100 µL aliquots at −80 °C until required.

### 4.14. Total Protein Determination

Pierce BCA (Bicinchoninic acid) Protein Assay Kit determined the total protein concentration (23225, Thermo Fisher Scientific, Waltham, MA, USA). The color formation was analyzed at 562 nm using a microplate reader (Multiskan GO, Thermo Fisher Scientific, Waltham, MA, USA).

### 4.15. Determination of Biomarker Expression by ELISA

The proline content was measured by using commercial colorimetric kit (A107, Nanjing Jiancheng Bioengineering Institute, Nanjing, China) or target-specific ELISA kits (ZGB-E1594, Zgenebio Inc., Taipei, Taiwan). The five extracellular matrix markers of hydroxyproline, chondroitin sulfate, dermatan sulfate, heparan sulfate, and keratan sulfate were measured by commercial target-specific ELISA kits (ZGB-E1591, ZGB-E1663, ZGB-E1664, ZGB-E1665, ZGB-E1666, Zgenebio Inc., Taipei, Taiwan). ROS (by measuring H_2_O_2_ level) was measured by commercial target-specific ELISA kit (ZGB-E1561, Zgenebio Inc., Taipei, Taiwan). The tissue anti-oxidative capacity markers of catalase and SOD were measured by commercial target-specific ELISA kits (ZGB-E1598 and ZGB-E1604, Zgenebio Inc., Taipei, Taiwan). A commercial colorimetric kit measured the total anti-oxidative capacity (T-AOC) (A015, Nanjing Jiancheng Bioengineering Institute, Nanjing, China). Commercial target-specific ELISA kits measured the stress hormone of cortisol (ZGB-E1575, Zgenebio Inc., Taipei, Taiwan). Some neurotransmitters, such as dopamine, GABA, serotonin, melatonin, norepinephrine, epinephrine, acetylcholine, acetylcholine esterase, glutamate, glycine, and histamine were estimated by commercial target-specific ELISA kits (ZGB-E1573, ZGB-E1574, ZGB-E1572, ZGB-E1597, ZGB-E1571, ZGB-E1589, ZGB-E1589, ZGB-E1585, ZGB-E1637, ZGB-E1588, ZGB-E1587, and ZGB-E1586, Zgenebio Inc., Taipei, Taiwan). The tissue energy markers of creatine kinase, ATP and CoQ10, were measured by commercial target-specific ELISA kits (ZGB-E1646, ZGB-E1645, ZGB-E1599, Zgenebio Inc., Taipei, Taiwan). The telomerase activity was measured by commercial target-specific ELISA kit (ZGB-E1596, Zgenebio Inc., Taipei, Taiwan). The target-specific ELISA kits used in this study were based on Sandwich ELISA principle. First, the target-specific antibodies were immobilized onto 96-well microplates. Later, the tissue homogenates and HRP (horseradish peroxidase)-conjugated target-specific antibodies were applied onto microplate and then incubated at 37 °C for 1 h. After washing with washing buffer, chromogen A and B were applied onto microplate and incubated at 37 °C for 15 min. Finally, stop solution was applied to stop color development and the absorbance was analyzed at 450 nm while using a microplate reader (Multiskan GO, Thermo Fisher Scientific, Waltham, MA, USA). The relative concentration of target protein was then quantified by comparing to the standard curve that is generated from the standard provided by commercial kits.

### 4.16. Real Time RT-PCR

Total RNA from different tissues of adult zebrafish that were aged at around six-month-old were extracted by the RNAZol^®^RT (Invitrogen) and quantified with microplate reader (Multiskan GO, Thermo Fisher Scientific, Waltham, MA, USA) at OD260 nm. RevertAid first cDNA synthesis kit (K1622, Fermentas, Waltham, MA, USA) was used to synthesize first-strand cDNA from total zebrafish RNA, according to the manufacturer’s instructions. RT-qPCR was performed using iQ SYBR Green Supermix (Bio-Rad Laboratories, Hercules, CA, USA) on a MyGO Pro (IT-IS Life Science Ltd., Mahon, Republic of Ireland) using *β-actin* as control, and the data were analyzed using the ∆∆Ct method [72]. The primer sequences used to perform quantitative real-time PCR for β-actin are forward: 5′-ATTGCTGACAGGATGCAGAAG-3′ and reversed: 5′-GATGGTCCAGACTCATCGTACTC-3′, and for *pycr1* gene are forward: 5′-GCCTCATATCATTCCCTTTGTCC-3′ and reversed: 5′-CTGCAGCAGTTTCTTCTCGA-3′.

## Figures and Tables

**Table 1:** Expression of neurotransmitters in the brain and other body parts in control and *pycr1* KO zebrafish at adult stage.

Biomarker	WT	*pycr1* KO	Unit	Significance	*p* Value
**Brain**					
Dopamine	0.282 ± 0.027	0.388 ± 0.086	pg/μg total protein	NO	0.3034
GABA	171.000 ± 13.930	235.700 ± 48.200	pg/μg total protein	NO	0.2671
5-HT	3.862 ± 0.432	5.553 ± 1.264	pg/μg total protein	NO	0.2742
Melatonin	13.170 ± 0.232	20.220 ± 4.245	pg/mg total protein	NO	0.1727
Norepinephrine	1.564 ± 0.071	2.281 ± 0.492	pg/μg total protein	NO	0.2224
Epinephrine	3.274 ± 0.166	5.178 ± 1.145	pg/μg total protein	NO	0.1751
Cortisol	1.253 ± 0.144	1.944 ± 0.613	pg/μg total protein	NO	0.3339
Acetylcholine	473.700 ± 23.370	678.800 ± 133.400	pg/μg total protein	NO	0.2044
Acetylcholinesterase	21.470 ± 0.947	30.530 ± 4.843	pg/μg total protein	NO	0.1402
Glutamate	31.870 ± 2.765	47.340 ± 7.596	pg/μg total protein	NO	0.1283
Glycine	27.820 ± 1.232	37.770 ± 8.194	μg/μg total protein	NO	0.2962
Histamine	8.295 ± 0.360	11.970 ± 2.025	pg/μg total protein	NO	0.1484
**Body**					
Dopamine	128.800 ± 19.520	65.790 ± 9.745	pg/μg total protein	YES	0.0136
GABA	77.630 ± 12.480	37.780 ± 5.180	pg/μg total protein	YES	0.0122
5-HT	1.854 ± 0.306	0.977 ± 0.114	pg/μg total protein	YES	0.0200
Melatonin	23.200 ± 4.166	12.870 ± 1.486	pg/mg total protein	YES	0.0376
Norepinephrine	0.653 ± 0.120	0.316 ± 0.030	pg/μg total protein	YES	0.0184
Epinephrine	2.122 ± 0.319	0.807 ± 0.115	pg/μg total protein	YES	0.0022
Cortisol	0.672 ± 0.096	0.299 ± 0.0419	pg/μg total protein	YES	0.0038
Acetylcholine	84.260 ± 11.860	48.320 ± 6.940	pg/μg total protein	YES	0.0225
Acetylcholinesterase	8.596 ± 1.636	4.097 ± 0.498	pg/μg total protein	YES	0.0220
Glutamate	4.121 ± 0.764	1.898 ± 0.267	pg/μg total protein	YES	0.0177
Glycine	32.620 ± 4.556	13.820 ± 1.194	μg/μg total protein	YES	0.0018
Histamine	1.466 ± 0.263	0.706 ± 0.089	pg/μg total protein	YES	0.0181

## Supplementary Materials

The following are available online at https://www.mdpi.com/2073-4409/8/5/453/s1. Video S1. Locomotion of wild type (left) and *pycr1* KO (right) zebrafish in novel tank test. Locomotion was recorded and analyzed by idTracker software. The upper chamber space was highlighted in yellow color. This video was played at 5× speed. Video S2. Locomotion of wild type (left) and *pycr1* KO (right) zebrafish in the predator avoidance test. The predator was placed in the left side and zebrafish is placed in the right side. A transparent glass separator was placed at 15 cm away from the vertical side wall to separate zebrafish and the predator. The predator approaching zones is highlighted in yellow color. This video is played at 10× speed. Video S3. Locomotion of wild type (left) and *pycr1* KO (right) zebrafish in the mirror biting test. The mirror was positioned to the right side of the wall. The mirror approaching zones is highlighted in yellow color. This video is played at 10× speed. Video S4. Locomotion of wild type (left) and *pycr1* KO (right) zebrafish in social interaction test. The normal zebrafish is placed in the left side and tested KO fish is placed in the right site and one transparent glass plate is inserted to separate both animals. The social interaction approaching zones are highlighted in yellow color. This video is played at 10× speed. Video S5. Locomotion of three wild type (left) and *pycr1* KO (right) zebrafish in shoaling test. This video was played at 10× speed.
