# Supplementary material for: Zebrafish Carrying pycr1 Gene Deficiency Display Aging and Multiple Behavioral Abnormalities
Source: Cells. 2019 May 14;8(5):453. doi: 10.3390/cells8050453 (PMC6562453; doi:10.3390/cells8050453)
Supplement: Supplementary file 1 [file cells-08-00453-s001.zip › supplementary data/Supplementray protocol_Zebrafish morphometric analysis protocol.pdf]

Zebrafish morphometric analysis protocol

Supplementary protocol for

Zebrafish carrying pycr1 gene deficiency display aging and multiple behavioral abnormalities

Sung-Tzu Liang [1], Gilbert Audira [1,2], Stevhen Juniardi [1], Jung-Ren Chen [3], Yu-Heng Lai [4], Zheng-Cai Du [5*], Dar-Shong Lin [6,7,8*] and **Chung-Der Hsiao** [1,2,9,10*]

1. Department of Bioscience Technology, Chung Yuan Christian University, Chung-Li, 32023, Taiwan
2. Department of Chemistry, Chung Yuan Christian University, Chung-Li, 32023, Taiwan
3. Department of Biological Science & Technology College of Medicine, I-Shou University, Kaohsiung, Taiwan
4. Department of Chemistry, Chinese Culture University, Taipei, 11114, Taiwan
5. Guangxi Scientific Experimental Center of Traditional Chinese Medicine, Guangxi University of Chinese Medicine, Nanning 530200 Guangxi, China;
6. Department of Pediatrics, Mackay Memorial Hospital, Taipei, Taiwan
7. Department of Medical Research, Mackay Memorial Hospital, Taipei, Taiwan
8. Department of Medicine, Mackay Medical College, New Taipei, Taiwan
9. Center for Biomedical Technology, Chung Yuan Christian University, Chung-Li, 32023, Taiwan
10. Center for Nanotechnology, Chung Yuan Christian University, Chung-Li, 32023, Taiwan

* Correspondence:
duzhengcai8@163.com (Z.-C.D.); dslin@ms1.mmh.org.tw (D.-S.L.); **cdhsiao@cycu.edu.tw (C.-D.H.)**

Analysis pipeline outline

1. Convert image file to .tps format using TpsUtil
2. Digitize the image landmark using TpsDig2
3. Procrustes analysis using MorphoJ
4. Generate covariance matrix using MorphoJ
5. Principal Component Analysis (PCA) using MorphoJ

1. Convert image file to .tps format using TpsUtil

- Download tpsUtil64 to convert image file to TPS file
  http://life.bio.sunysb.edu/morph/soft-utility.html

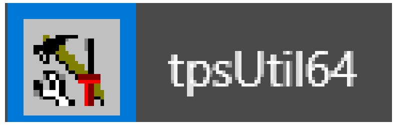

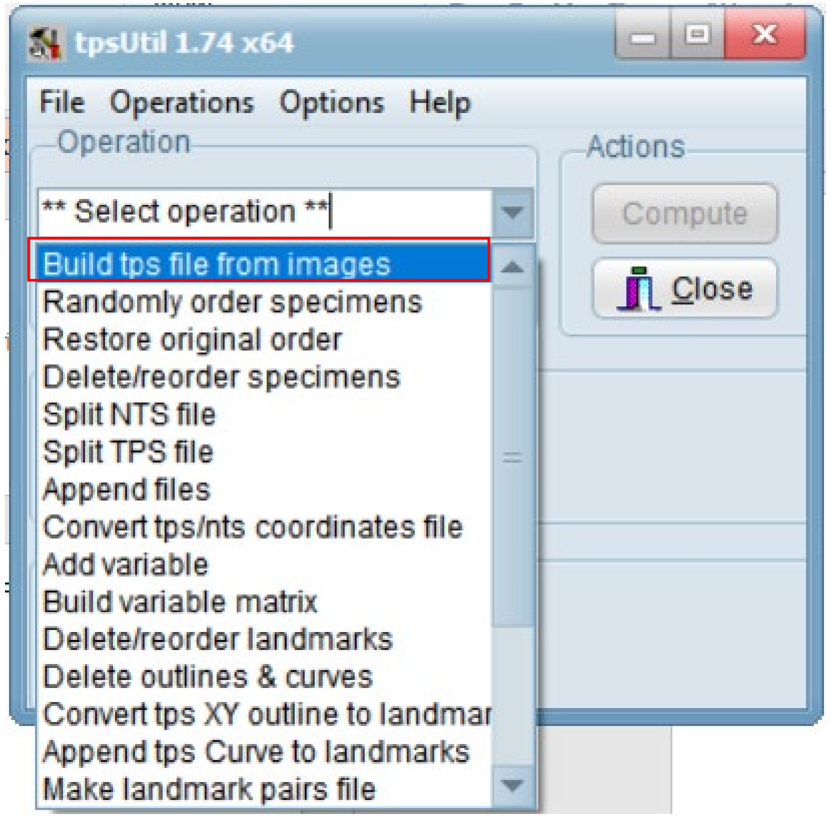

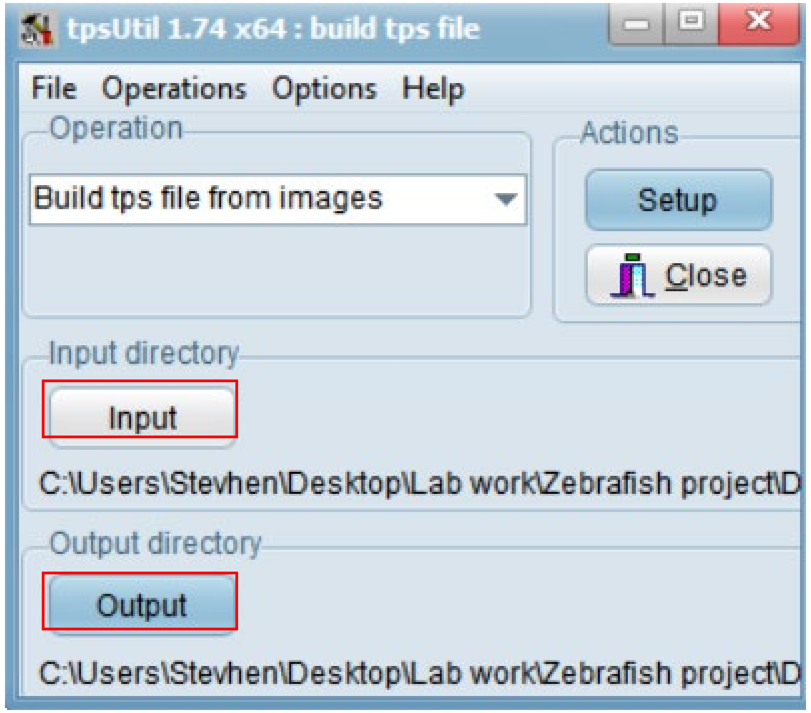

Select the Input and Output Directory

1. Convert image file to .tps format using tpsUtil

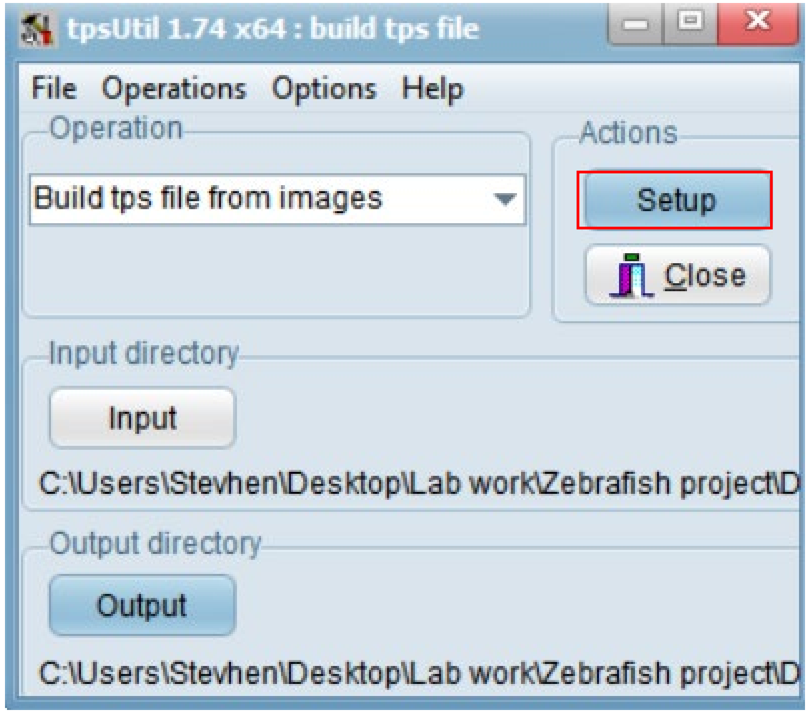

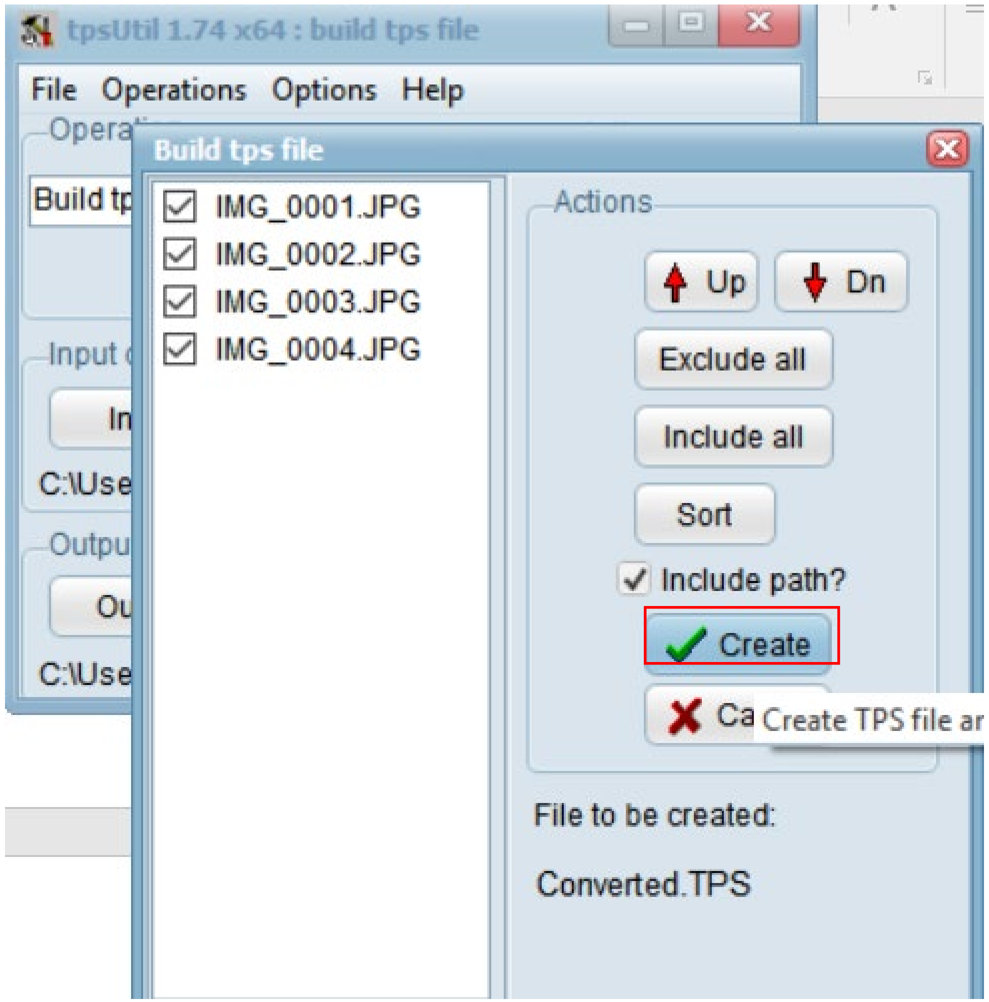

Select all the image to be measured in the TPS file and create the file

2. Digitize the image landmark using TpsDig2

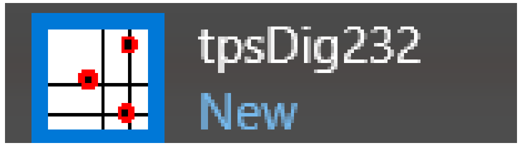

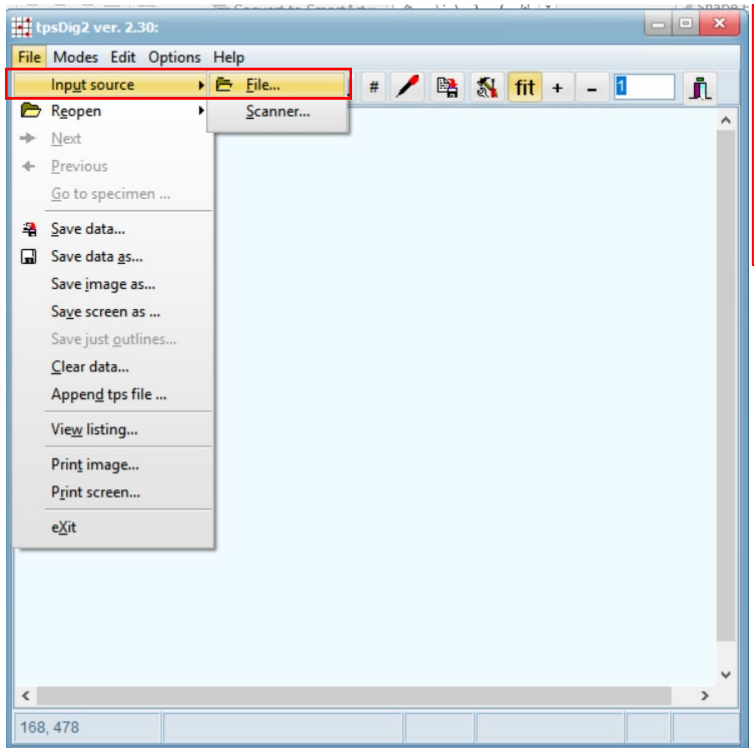

Open the .tps file by selecting the input source

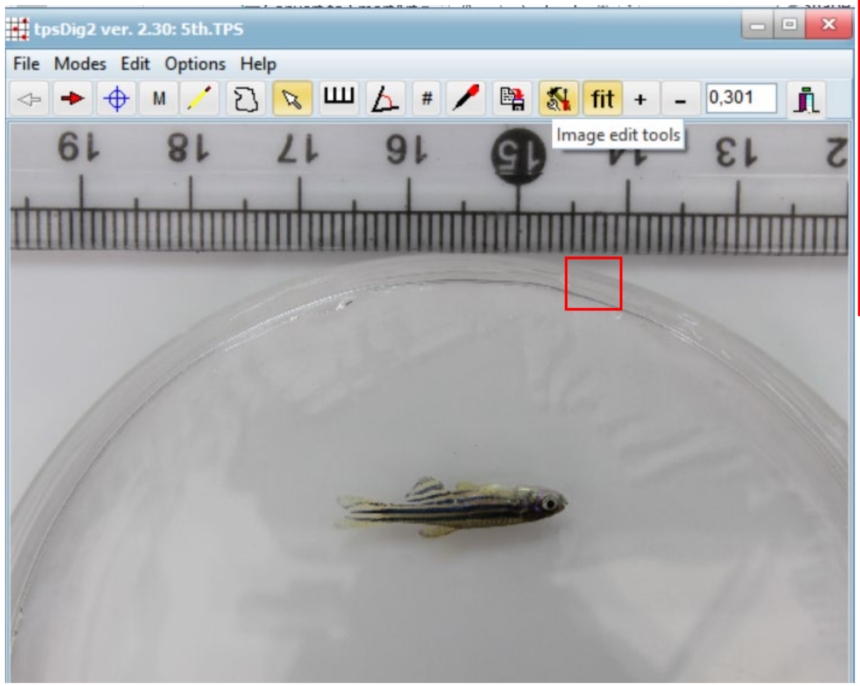

Edit the image using the edit tools image and set the scale

2. Digitize the image landmark using TpsDig2

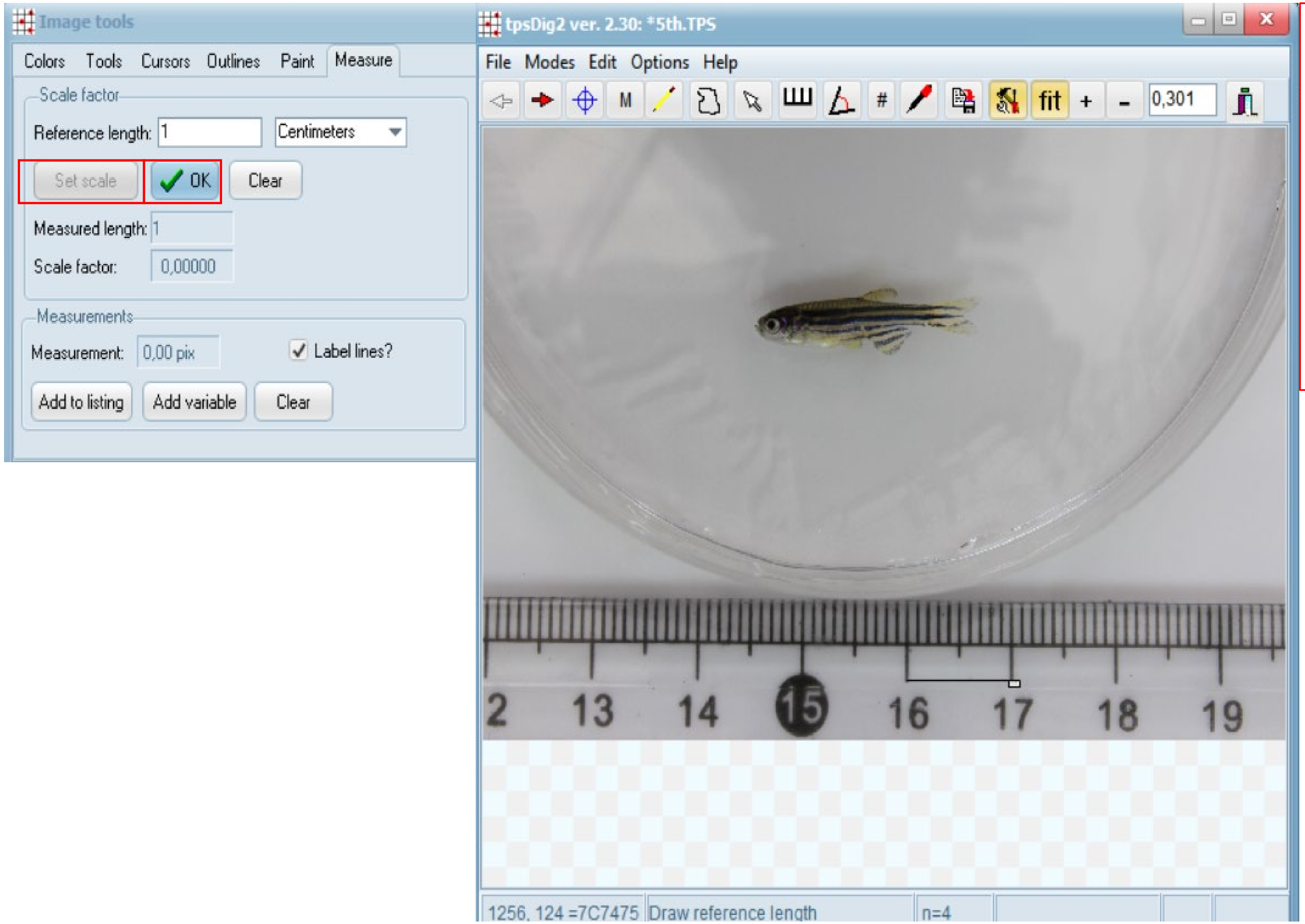

Set the scale by selecting the measure tab and press the OK button

2. Digitize the image landmark using TpsDig2

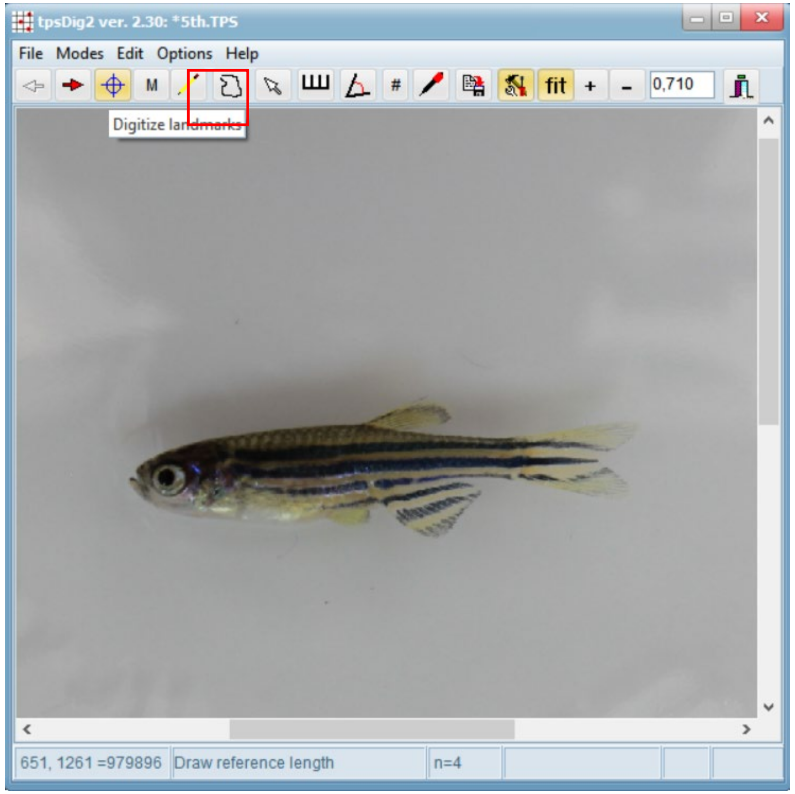

Select the digitize landmarks icon then click the landmark

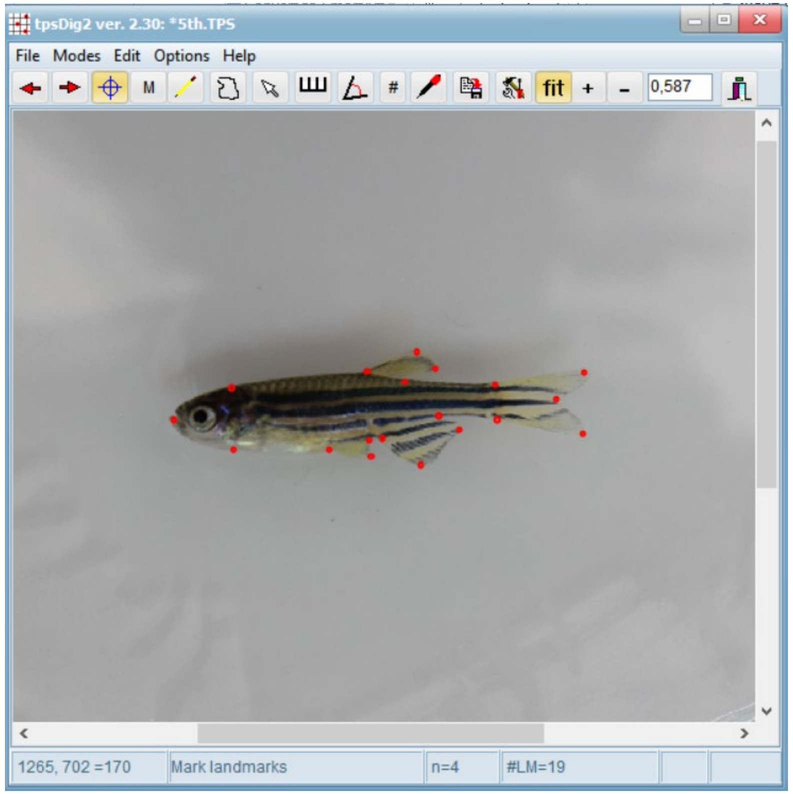

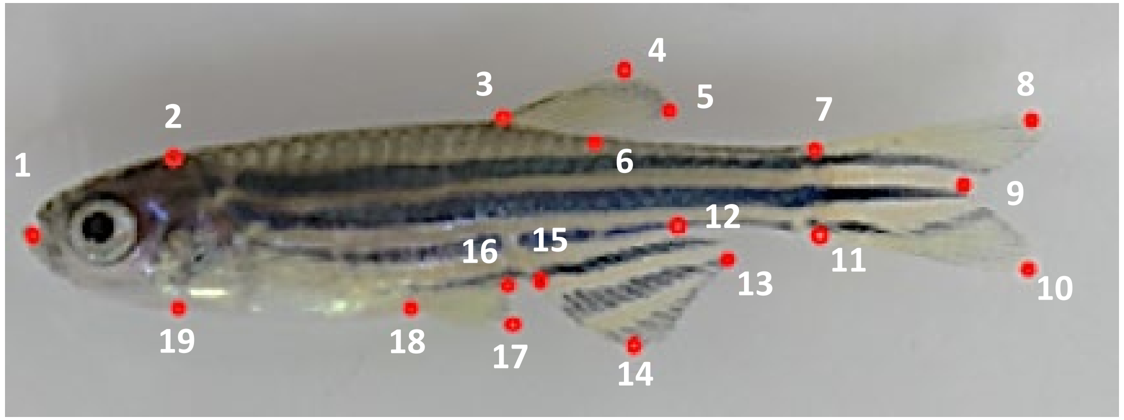

In our case,
19 landmarks were selected

2. Digitize the image landmark using TpsDig2

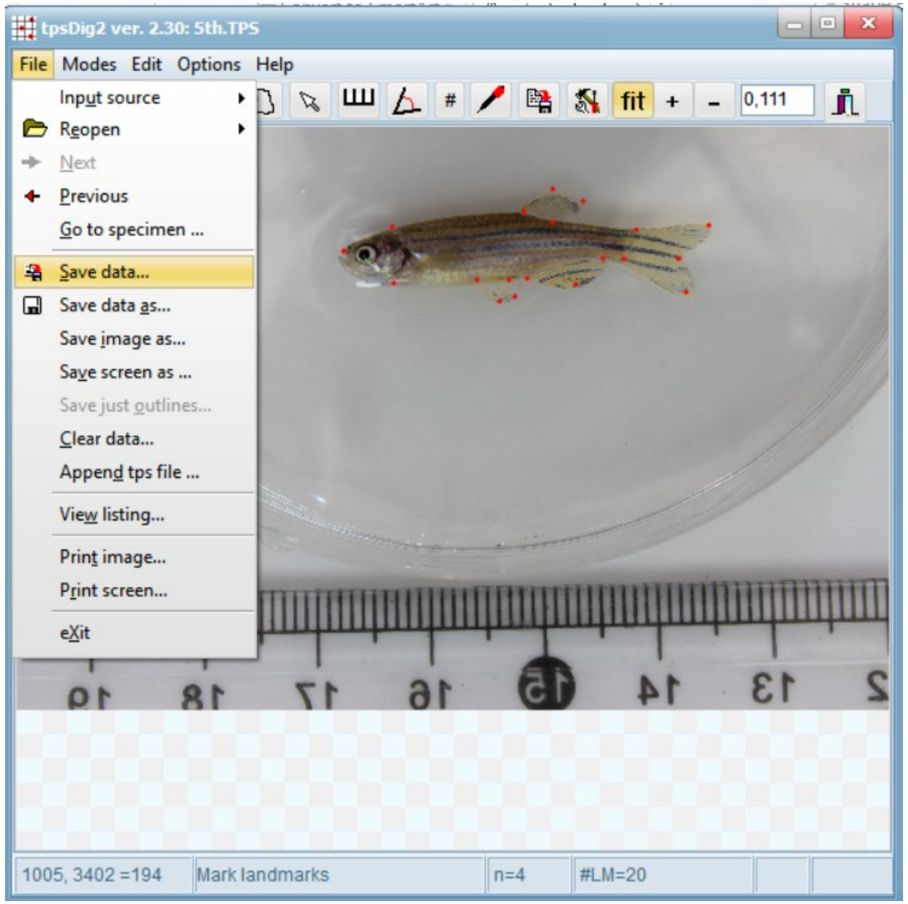

Save the file in .TPS after digitizing the landmark

To do the Principal component analysis (PCA), we need to install **MorphoJ** program

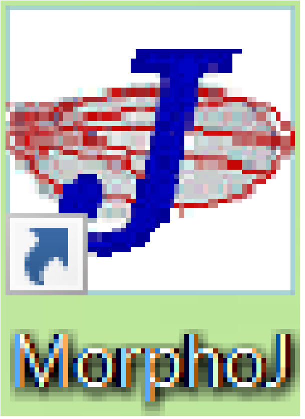

MorphoJ is an open-source program and can be downloaded freely http://www.flywings.org.uk/morphoj_page.htm

3. Procrustes analysis using MorphoJ

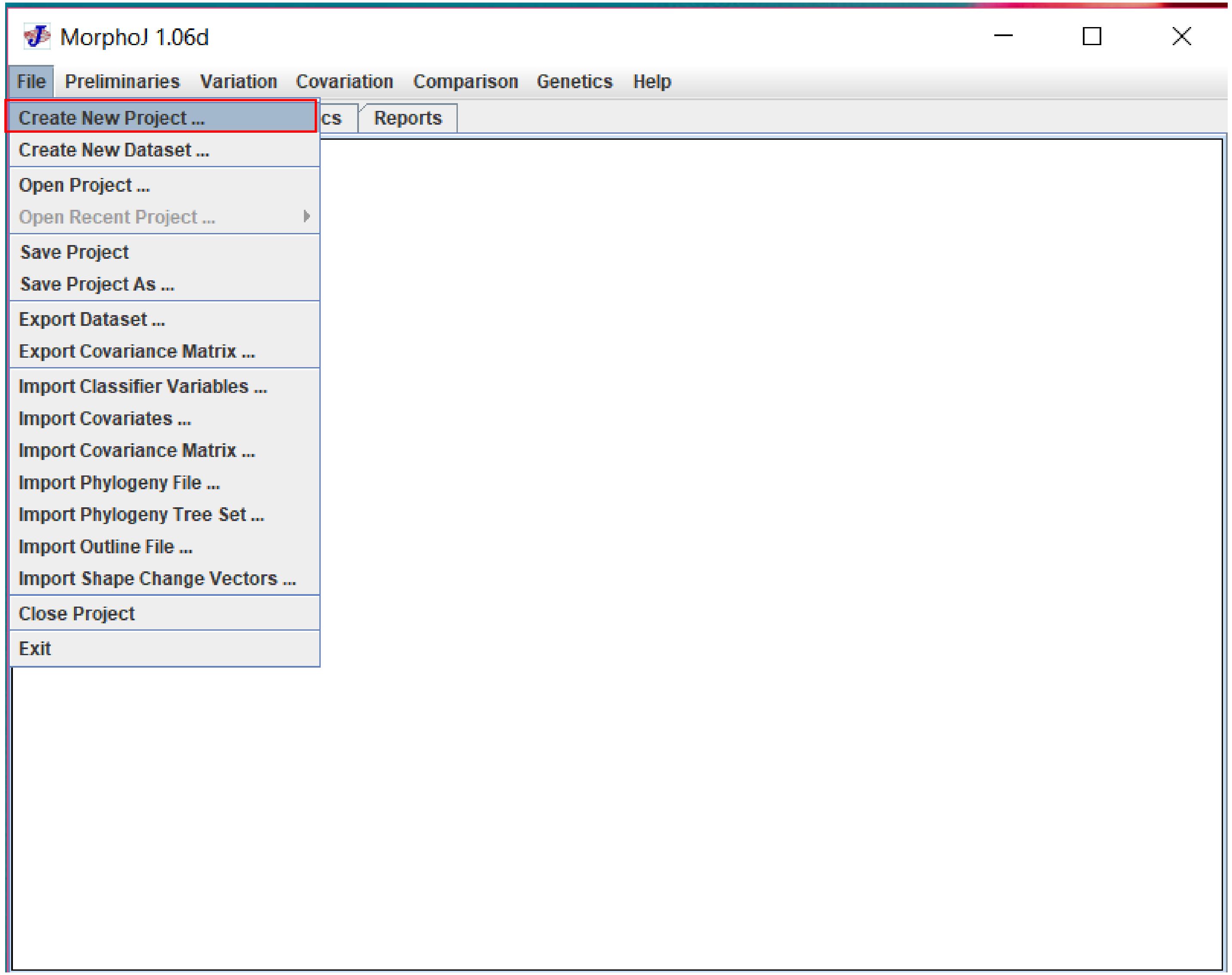

Create New Project by choosing the File Tab

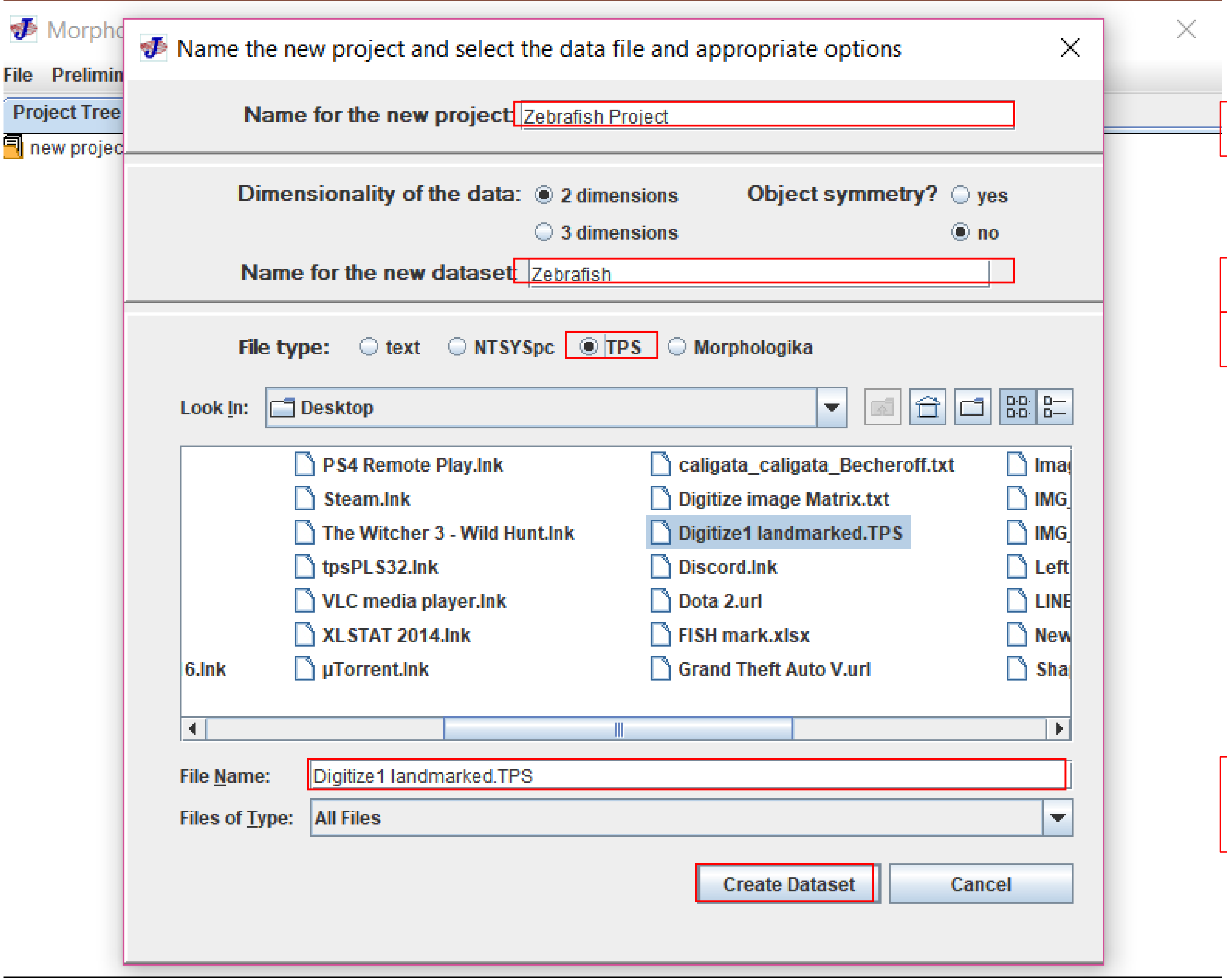

Put a name for the project

Put a name for the dataset

Select the data file type

Select the .tps from tpsDig2 output

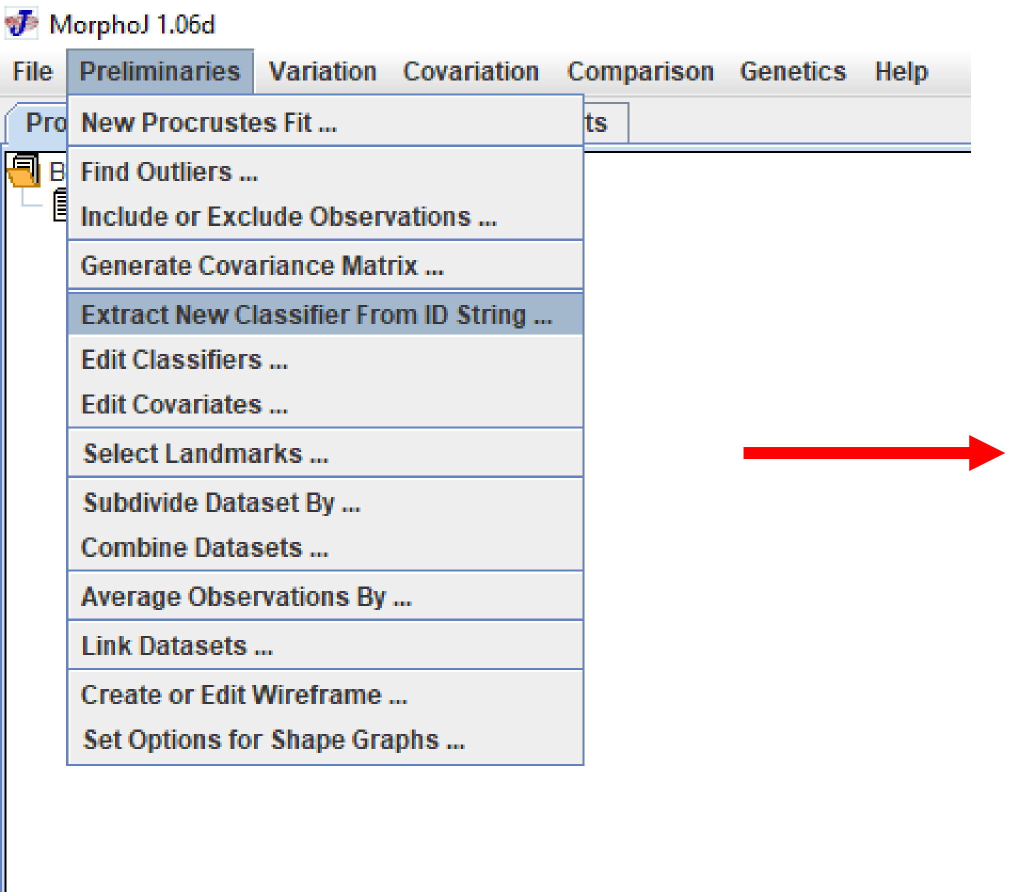

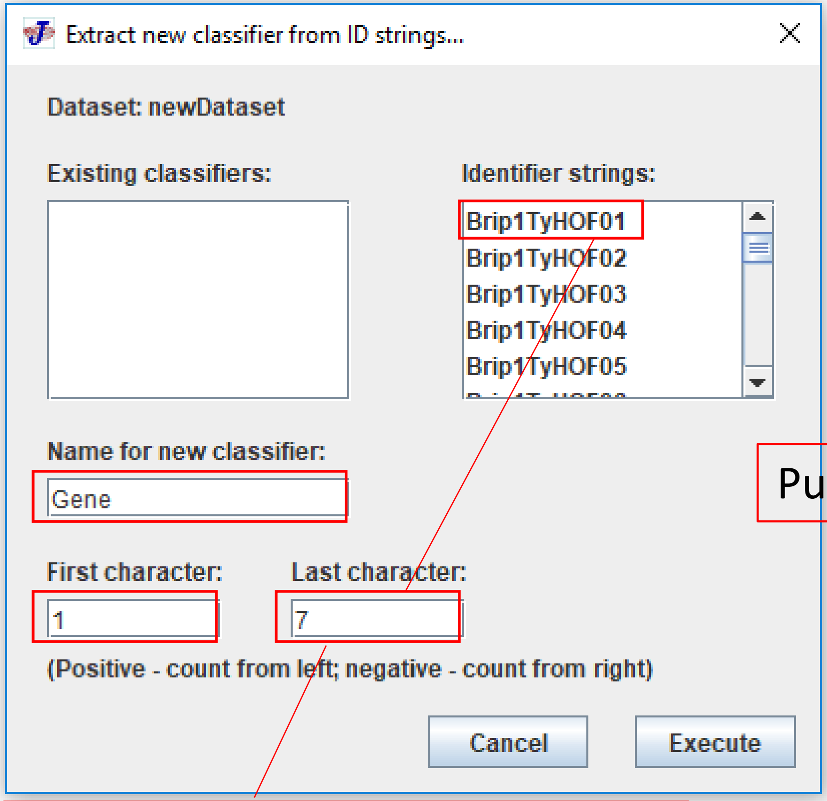

Put the name for a new classifier

For gene name start from 1-7 in this example

Select the newdatasetTo make a new classifier/can divide the sample by gender/gene

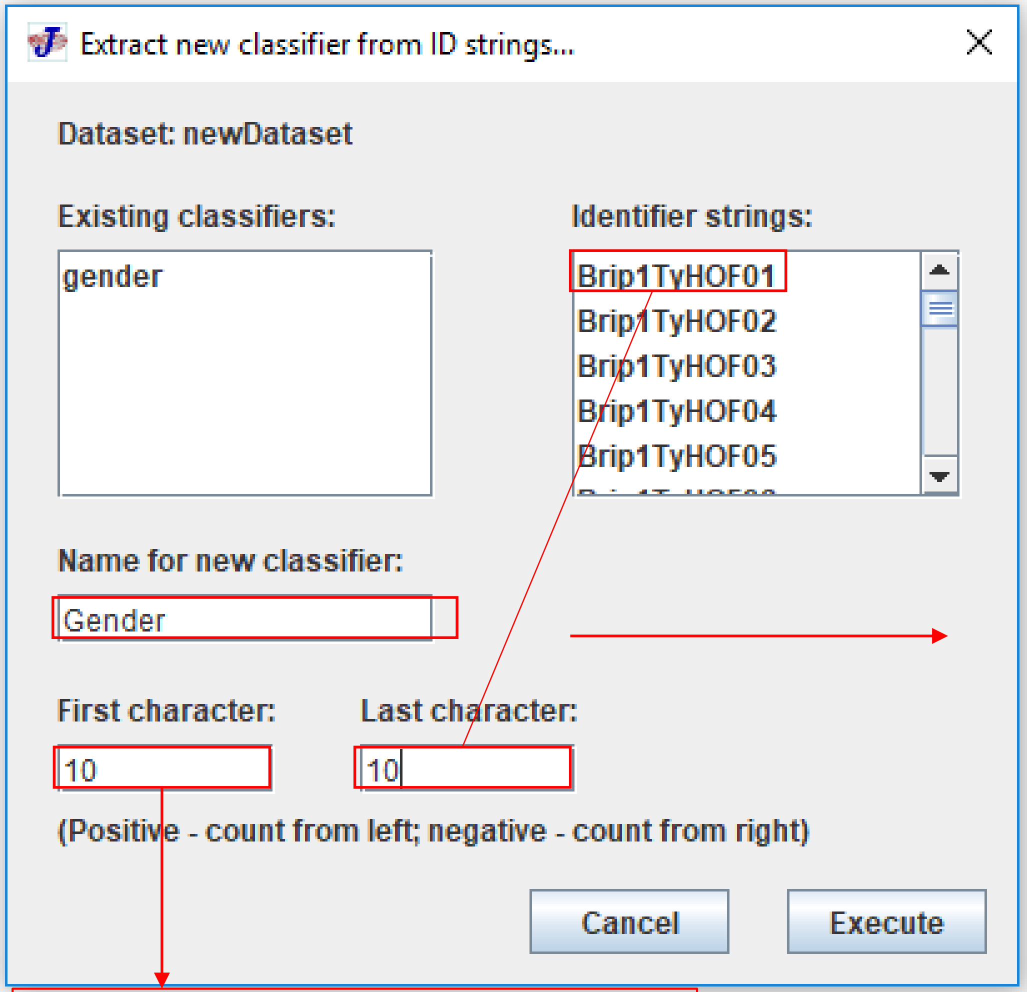

Put the name for a new classifier

For Gender name start from 10 to 10 in this example

3. Procrustes analysis using MorphoJ

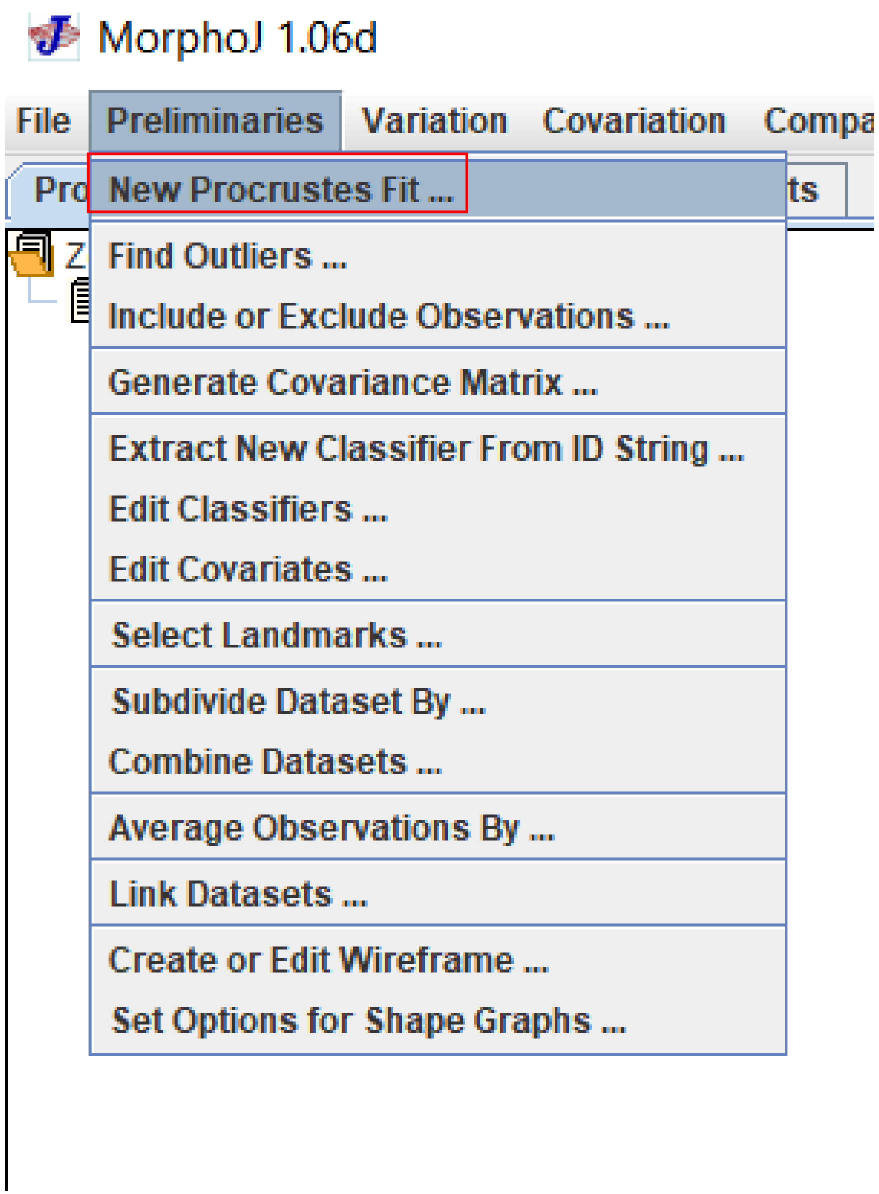

Do Procrustes fit analysis to analyse the distribution set of shape

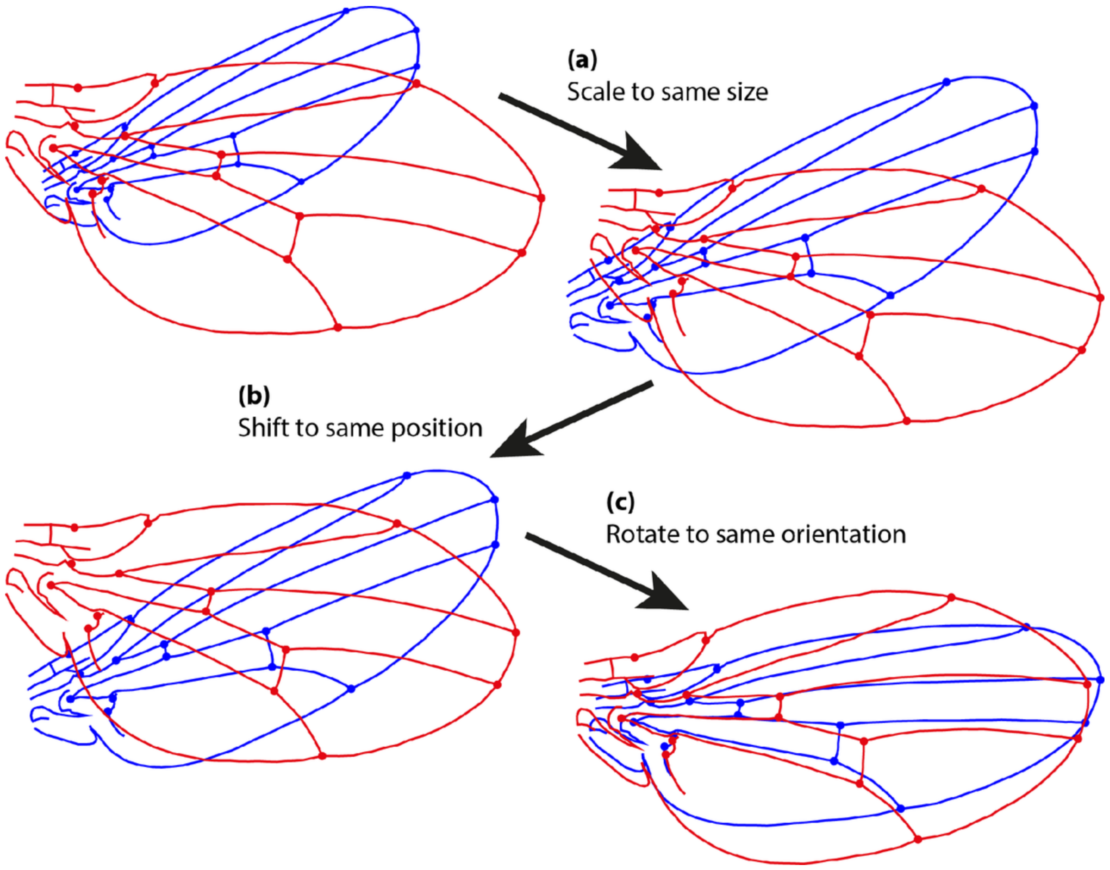

3. Procrustes analysis using MorphoJ

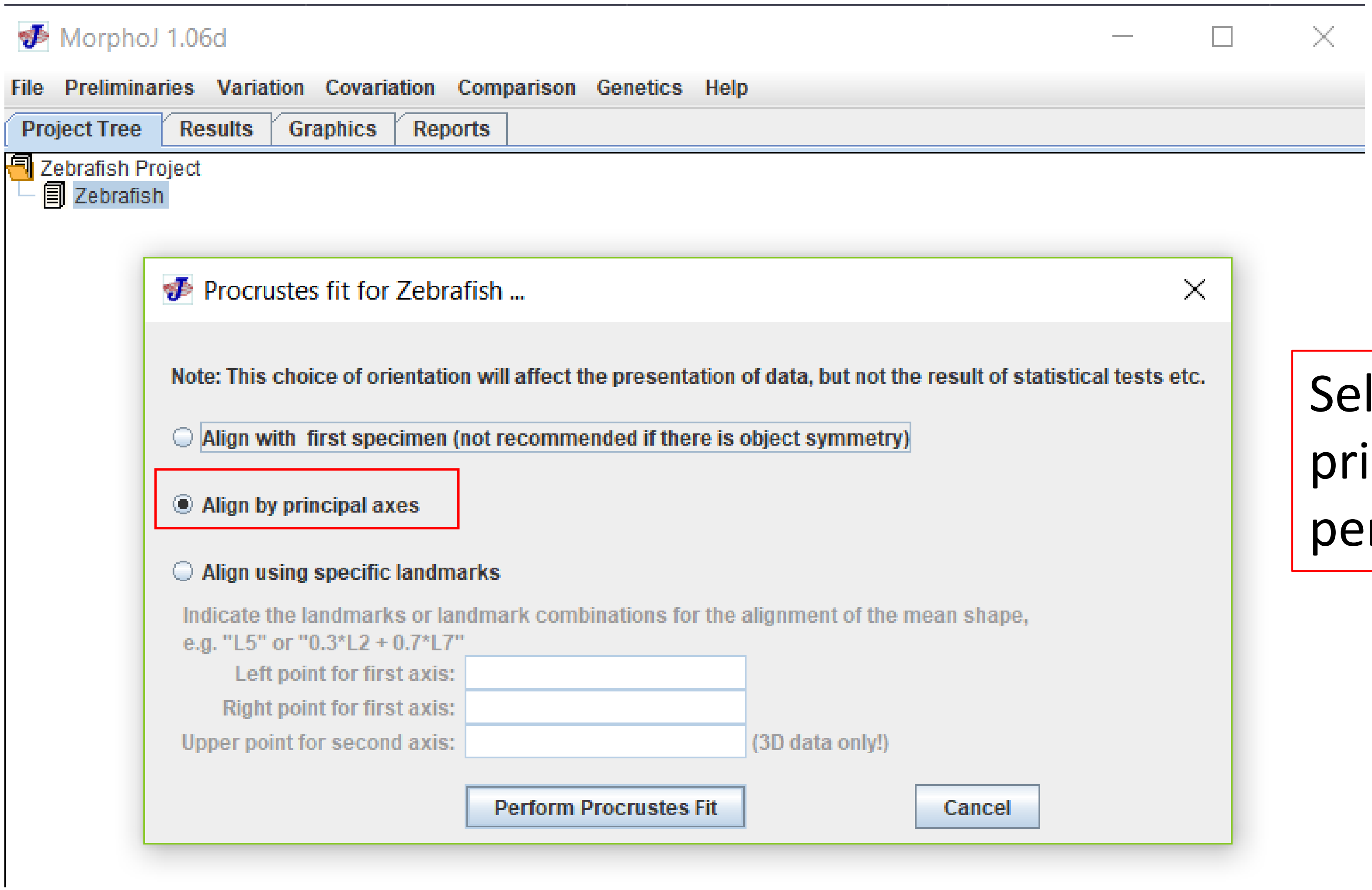

Select the Align by principal axes then perform **Procrustes Fit**

3. Procrustes analysis using MorphoJ

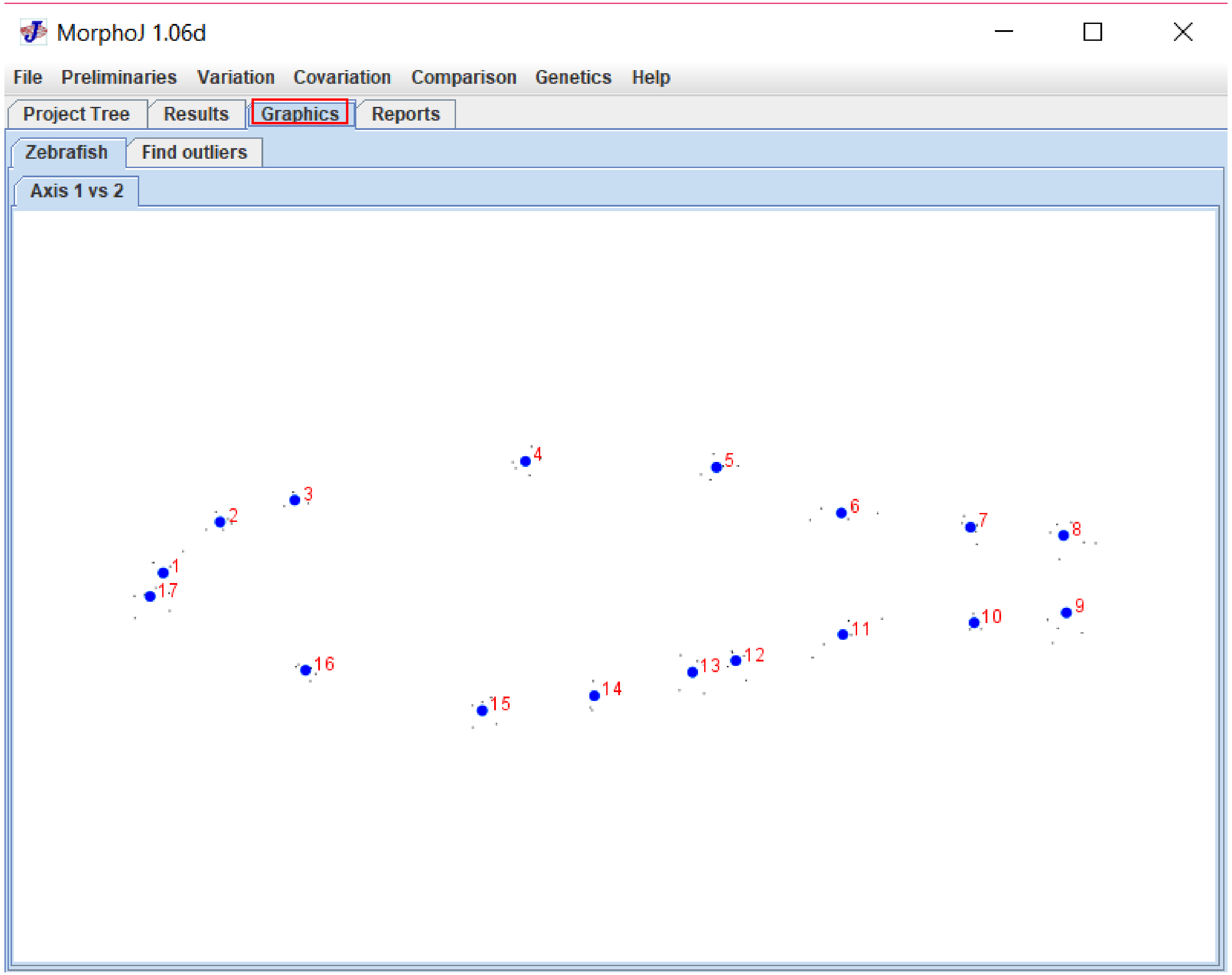

Procrustes Fit analyses result can be viewed in graphics tab

In this case,
17 landmarks were selected

4. Generate covariance matrix using MorphoJ

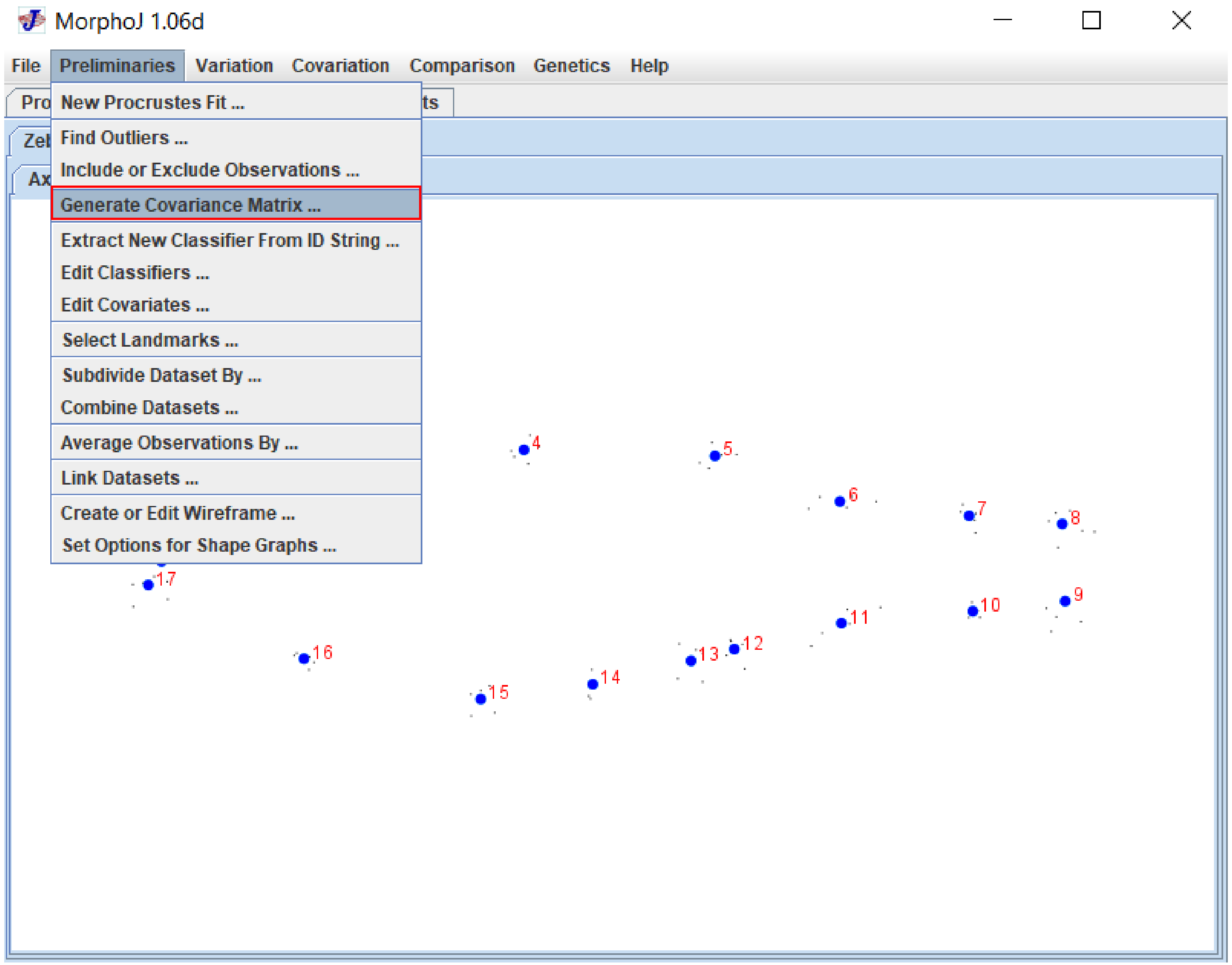

Generate **covariance matrix** by selecting the preliminaries tab

4. Generate covariance matrix using MorphoJ

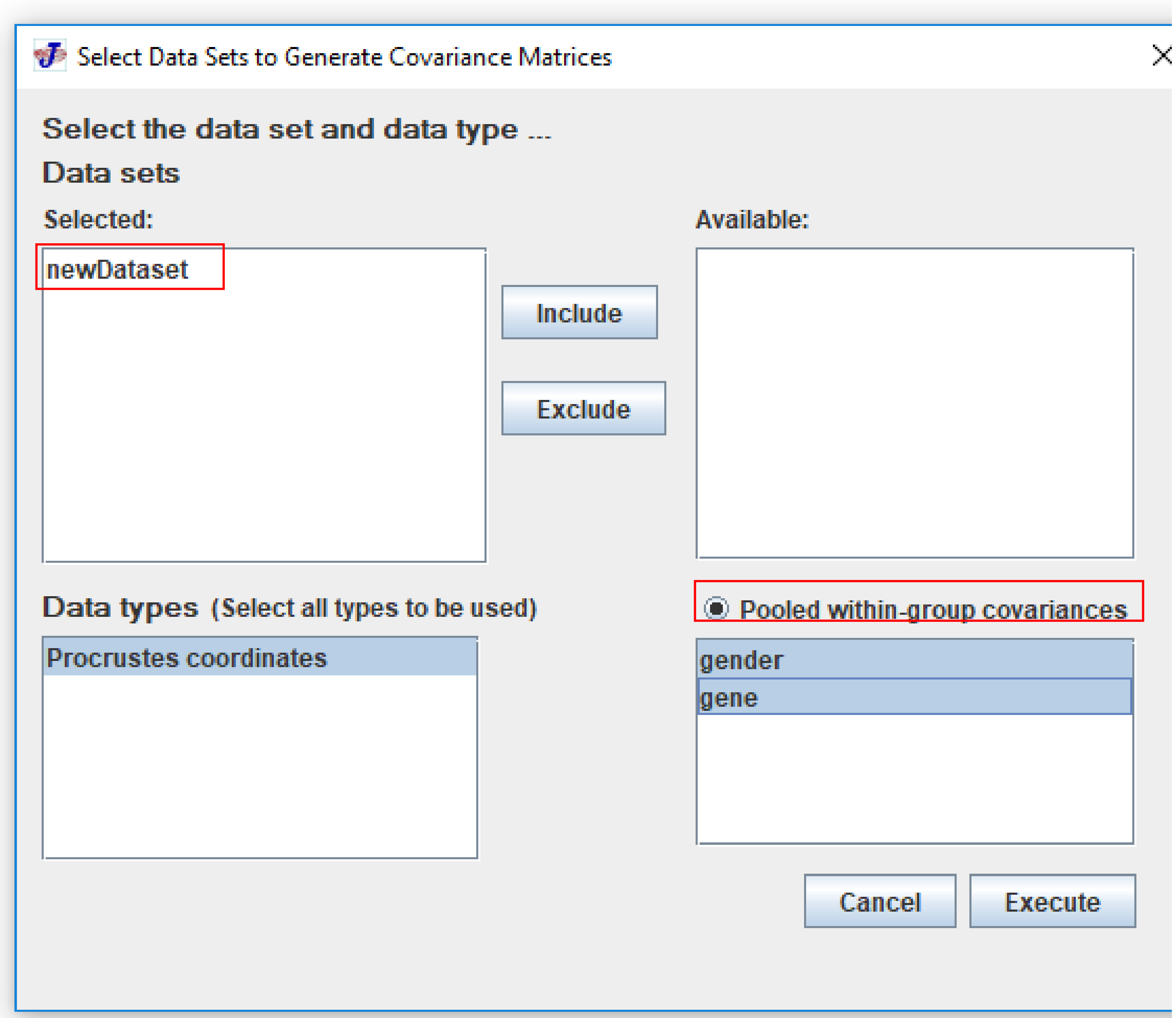

Select the dataset and "pooled within group covariance" then execute the program to generate **covariance matrices**

4. Generate covariance matrix using MorphoJ

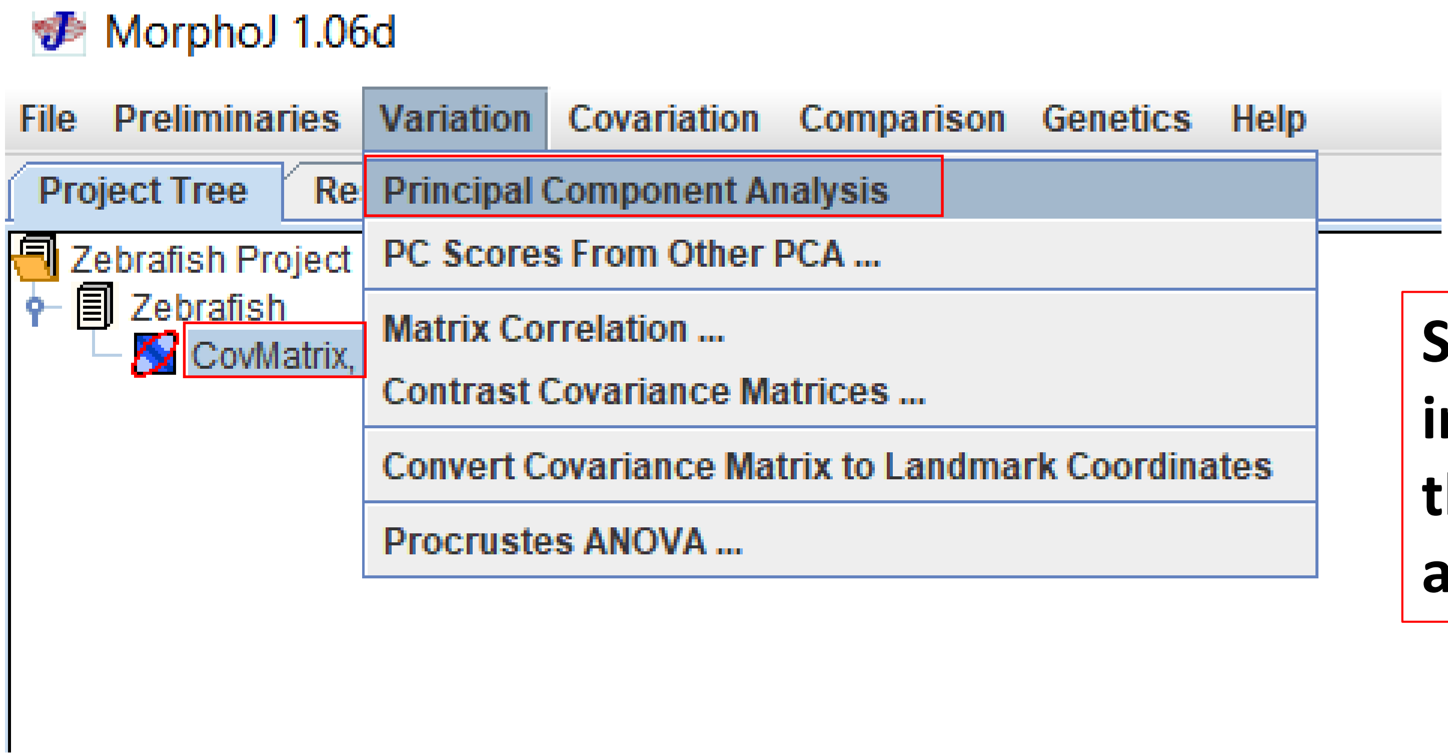

Select the covariance matrices in the project tree tab and do the principal component analysis in the variation tab

5. Principal Component Analysis (PCA) using MorphoJ

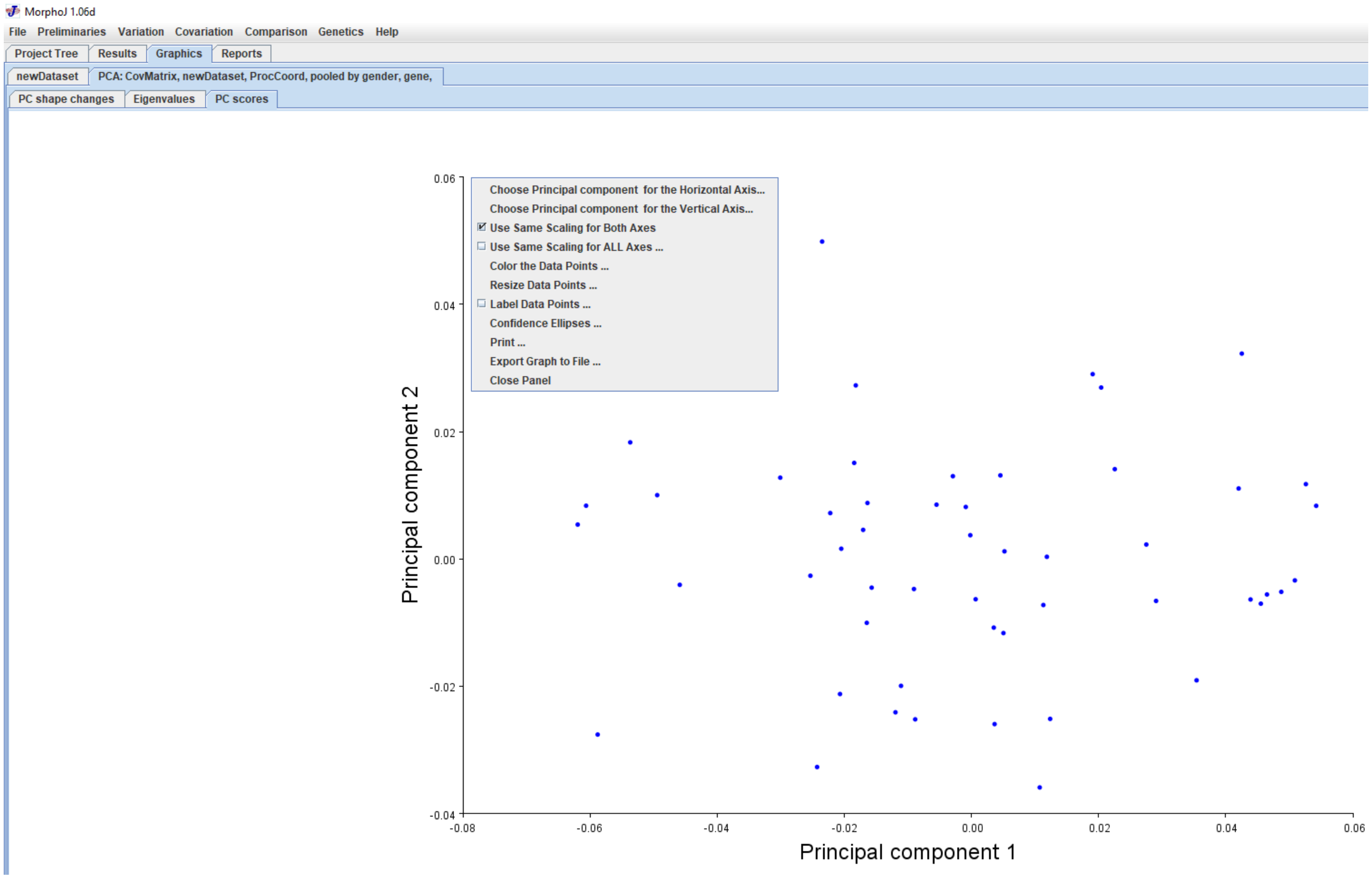

View the results in the graphics tab and click right in the image to edit several parameter

5. Principal Component Analysis (PCA) using MorphoJ

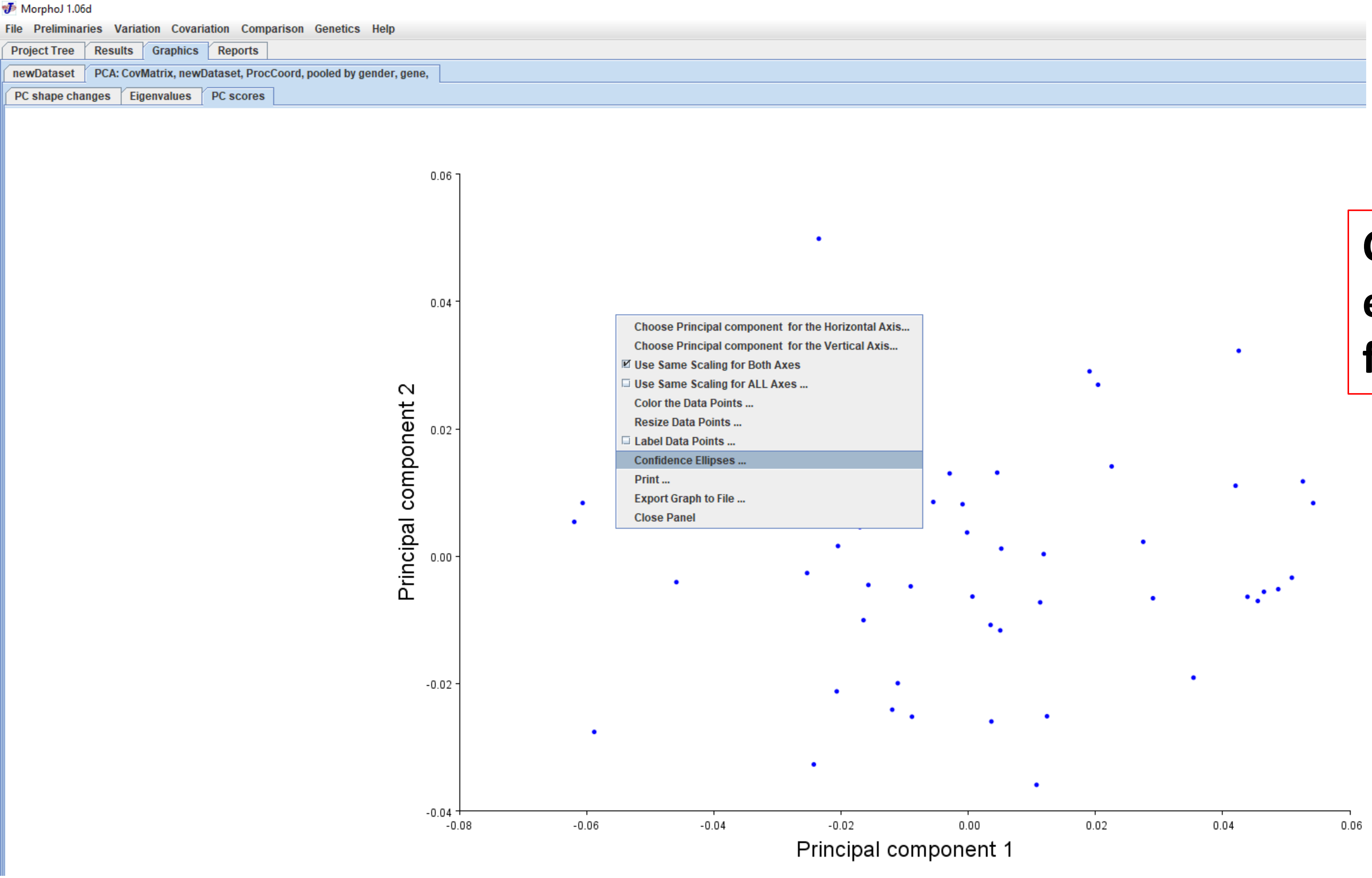

Choose Confidence ellipses to make ellipses from the data

5. Principal Component Analysis (PCA) using MorphoJ

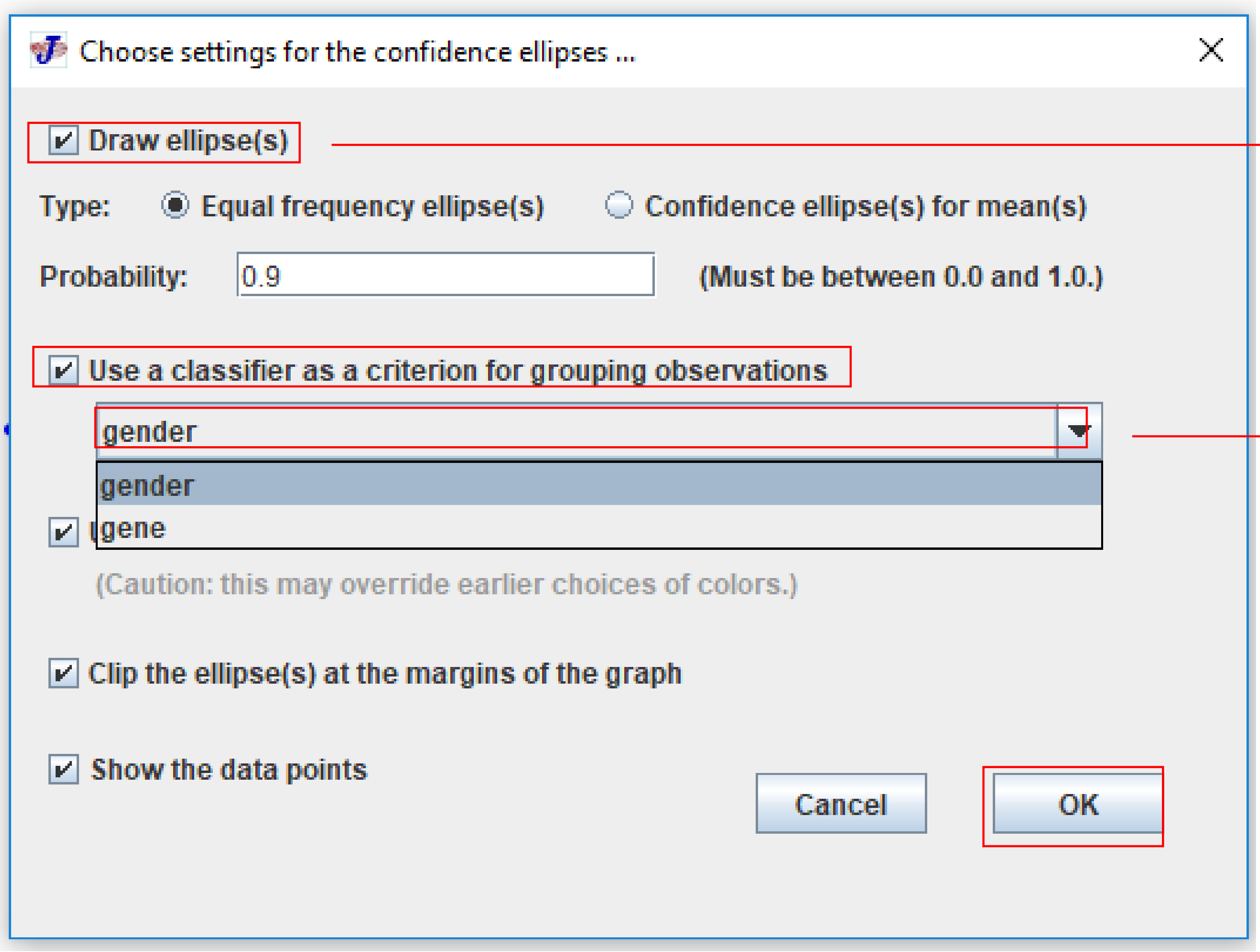

Choose Draw ellipses

Choose the category “Gene or Gender”

5. Principal Component Analysis (PCA) using MorphoJ

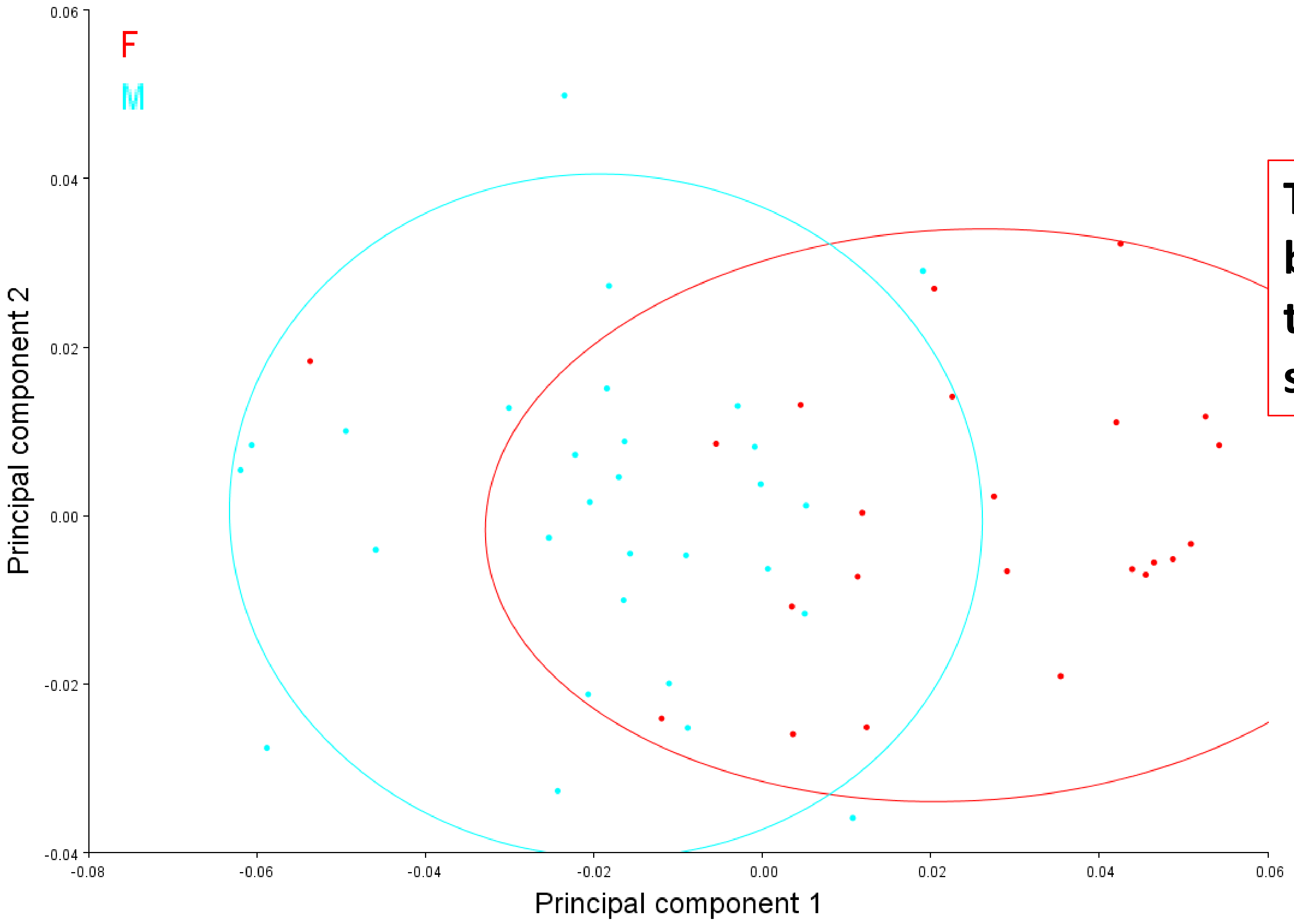

The ellipses graphic will be made like this from the sample and can be seen in the PC score tab.
